# Digitonin‐Loaded Nanoscale Metal–Organic Framework for Mitochondria‐Targeted Radiotherapy‐Radiodynamic Therapy and Disulfidptosis

**DOI:** 10.1002/adma.202405494

**Published:** 2024-09-10

**Authors:** Wenyao Zhen, Yingjie Fan, Tomas Germanas, Langston Tillman, Jinhong Li, Abigail L. Blenko, Ralph R. Weichselbaum, Wenbin Lin

**Affiliations:** ^1^ Department of Chemistry The University of Chicago Chicago IL 60637 USA; ^2^ Department of Radiation and Cellular Oncology and the Ludwig Center for Metastasis Research The University of Chicago Chicago IL 60637 USA

**Keywords:** disulfidptosis, GLUT1, immune checkpoint downregulation, metal–organic framework, radiotherapy‐radiodynamic therapy, xCT‐SLC7A11

## Abstract

The efficacy of radiotherapy (RT) is limited by inefficient X‐ray absorption and reactive oxygen species generation, upregulation of immunosuppressive factors, and a reducing tumor microenvironment (TME). Here, the design of a mitochondria‐targeted and digitonin (Dig)‐loaded nanoscale metal‐organic framework, Th‐Ir‐DBB/Dig, is reported to overcome these limitations and elicit strong antitumor effects upon low‐dose X‐ray irradiation. Built from Th_6_O_4_(OH)_4_ secondary building units (SBUs) and photosensitizing Ir(DBB)(ppy)_2_^2+^ (Ir‐DBB, DBB = 4,4′‐di(4‐benzoato)−2,2′‐bipyridine; ppy = 2‐phenylpyridine) ligands, Th‐Ir‐DBB exhibits strong RT‐radiodynamic therapy (RDT) effects via potent radiosensitization with high‐Z SBUs for hydroxyl radical generation and efficient excitation of Ir‐DBB ligands for singlet oxygen production. Th‐Ir‐DBB/Dig releases digitonin in acidic TMEs to trigger disulfidptosis of cancer cells and sensitize cancer cells to RT‐RDT through glucose and glutathione depletion. The released digitonin simultaneously downregulates multiple immune checkpoints in cancer cells and T cells through cholesterol depletion. As a result, Th‐Ir‐DBB/dig plus X‐ray irradiation induces strong antitumor immunity to effectively inhibit tumor growth in mouse models of colon and breast cancer.

## Graphical abstract

A mitochondria‐targeted cationic nanoscale metal organic framework shows strong radiotherapy‐radiodynamic therapy effects and selectively releases digitonin in acidic tumor microenvironments to induce disulfidptosis of cancer cells and downregulate immune checkpoints in cancer and T cells, thereby eliciting strong antitumor immunity to effectively inhibit tumor growth.

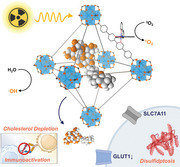

## 1 Introduction

High‐energy ionizing X‐ray radiation penetrates tissues deeply to deposit energy, generate reactive oxygen species (ROS), and induce DNA damage in tumors for cancer radiotherapy (RT).^[^ ^1^ ^]^ Besides eradicating local tumors, RT has been shown to occasionally control tumors beyond the irradiated zones by triggering an antitumor immune response (abscopal effect).^[^ ^2^ ^]^ As high doses of X‐rays are needed to achieve effective RT, which can cause debilitating side effects, significant efforts have been devoted to the development of nanomaterials containing high‐Z elements to augment radiation energy deposition, thereby enhancing the radiotherapeutic effects against deep‐seated tumors. However, the effectiveness of radio‐enhancement by nanomaterials has been limited by inefficient absorption of X‐rays, low efficiency in generating singlet oxygen (^1^O_2_) and other highly cytotoxic ROS, short lifetime and diffuse distance of ROS,^[^ ^3^ ^]^ limited immune responses of RT, and the inherent physiological barriers of the tumor microenvironment (TME).

We disclosed the ability of nanoscale metal‐organic frameworks (nMOFs) to enhance the radiotherapeutic effects of X‐rays in 2014^[^ ^4^ ^]^ and elucidated the radio‐enhancement mechanisms of nMOFs over the past few years.^[^ ^5^ ^]^ In particular, we uncovered efficient radio enhancement with nMOFs through a unique radiotherapy‐radiodynamic therapy (RT‐RDT) process via the simultaneous incorporation of high‐Z secondary building units (SBUs) and photosensitizing ligands. With their porous structures^[^ ^6^ ^]^ and periodically arranged high‐Z SBUs,^[^ ^7^ ^]^ nMOFs showed increased radio‐enhancement over other nanomaterials by enhancing energy deposition from X‐rays, promoting ^1^O_2_ generation, and facilitating ROS diffusion. The increased ^1^O_2_ generation in turn elicits a stronger immune response. However, it has not been explored if the porous structures of nMOFs can be used to deliver therapeutic molecules to overcome the physiological barriers of the TME.

The inherent barriers in the TME include hypoxia, highly expressed molecules or enzymes with antioxidant and pro‐tumor properties, dense extracellular matrix, among others.^[^ ^8^ ^]^ Tumor cells exhibit abnormally high accumulation of cholesterol due to increased receptor‐mediated endocytosis of cholesterol‐binding low‐density lipoproteins and elevated cholesterol synthesis from acetyl coenzyme A via the “mevalonate metabolism” pathway in the endoplasmic reticulum.^[^ ^9^ ^]^ Elevated levels of cholesterol in tumors cause T cell exhaustion characterized by increased expression of immune checkpoints such as cluster of differentiation 244 (CD244, also known as 2B4) and T‐cell immunoglobin and mucin domain‐3 (TIM‐3), leading to an immunosuppressive TME.^[^ ^10^ ^]^ Additionally, RT can elevate the secretion of immunosuppressive cytokines, including vascular endothelial growth factor, transforming growth factor‐beta, and interleukin‐10,^[^ ^11^ ^]^ upregulating immune checkpoints and other immune‐suppressive molecules, such as programmed cell death ligand 1 (PD‐L1)^[^ ^12^ ^]^ and indolamine‐2,3‐dioxygenase. These cytokines and immune checkpoints suppress immune cell function in the TME.^[^ ^13^ ^]^

Cancer cells have also developed strategies to become resistant to ROS. Solute carrier family 7 member 11 (xCT‐SLC7A11), a transporter for cystine, is frequently upregulated in cancer cells.^[^ ^14^ ^]^ Upon transport into the cytosol by xCT‐SLC7A11, cystine is converted to cysteine for glutathione (GSH) synthesis.^[^ ^15^ ^]^ High GSH levels in cancer cells can neutralize ROS^[^ ^16^ ^]^ to promote cell survival and proliferation.^[^ ^17^ ^]^ Inhibition of xCT‐SLC7A11‐mediated cystine transport with erastin has been shown to deplete intracellular GSH and induce ferroptotic cell death, primarily through lipid peroxidation in cell membranes.^[^ ^18^ ^]^ As the reduction of cystine to cysteine consumes an equivalent amount of glucose‐derived nicotinamide adenine dinucleotide phosphate (NADPH),^[^ ^14a^ ^]^ cancer cell proliferation is highly dependent on glucose. Recently, a new form of programmed cell death, disulfidptosis, was discovered in xCT‐SLC7A11^high^ cancer cells. Glucose depletion induced disulfidptosis in xCT‐SLC7A11^high^ cancer cells^[^ ^19^ ^]^ via accumulating excess disulfides, inducing disulfide bonding in the actin cytoskeleton and depleting NADPH, which further leads to actin network collapse, thereby increasing the vulnerability of cancer cells.^[^ ^20^ ^]^ Furthermore, xCT‐SLC7A11^high^ cancer cells are more sensitive to glucose depletion than xCT‐SLC7A11^low^ counterparts, and glucose transporter 1 (GLUT1) inhibition has been shown to induce disulfidptosis in xCT‐SLC7A11^high^ cancer cells.^[^ ^21^ ^]^ We surmised that nMOFs could be loaded with small molecules to deplete cholesterol and glucose in cancer cells to enhance the therapeutic effects of RT‐RDT.

**Figure 1:**
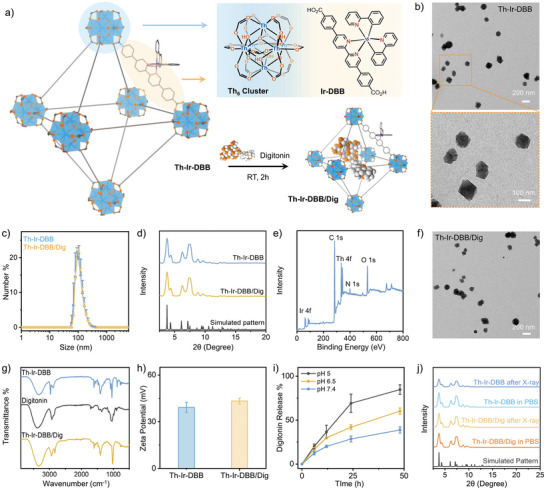
Preparation and characterization of Th‐Ir‐DBB/Dig. a) Synthesis of Th‐Ir‐DBB/Dig. b,f) TEM images of Th‐Ir‐DBB b) and Th‐Ir‐DBB/Dig f). c) DLS plots of Th‐Ir‐DBB and Th‐Ir‐DBB/Dig (*n* = 3). d) PXRD patterns of Th‐Ir‐DBB and Th‐Ir‐DBB/Dig. e) XPS spectrum of Th‐Ir‐DBB. g) FT‐IR spectra of digitonin, Th‐Ir‐DBB, and Th‐Ir‐DBB/Dig. h) Zeta potentials of Th‐Ir‐DBB and Th‐Ir‐DBB/Dig (*n* = 3). i) Release profiles of digitonin from Th‐Ir‐DBB/Dig in PBS solutions with different pH values (*n* = 3). j) Stability of Th‐Ir‐DBB and Th‐Ir‐DBB/Dig in PBS (1 mm) over 3 days or post X‐ray irradiation (12 Gy).

Here, we first designed a mitochondria‐targeted cationic nMOF, Th‐Ir‐DBB, with enhanced RT‐RDT efficacy over previously reported nMOFs. Th‐Ir‐DBB was constructed from Th_6_O_4_(OH)_4_ SBUs^[^ ^22^ ^]^ and Ir(DBB)(ppy)^2+^ [Ir‐DBB, DBB = 4,4′‐di(4‐benzoato)−2,2′‐bipyridine; ppy = 2‐phenylpyridine] photosensitizing ligands (**Figure** **1**a). Thorium (Th) has low radioactivity compared to other f‐elements, making it safe for biomedical applications, and, with a high atomic number (Z = 90), Th shows an enhanced ability to interact with ionizing radiation. The K‐edge energy (110 keV) of Th effectively interacts with high‐energy megavoltage radiation used in clinical settings, such as linear particle accelerators and ^60^Co sources.^[^ ^23^ ^]^ The high‐Z SBUs and highly photosensitizing Ir‐DBB ligands work synergistically to mediate highly efficient RT‐RDT. Th‐Ir‐DBB was further loaded with digitonin (Dig), a plant‐derived amphiphilic molecule that permeabilizes cell membranes and depletes cholesterol, to potentiate the antitumor efficacy of Th‐Ir‐DBB‐mediated RT‐RDT. Th‐Ir‐DBB/Dig showed tumor‐responsive release of digitonin in the acidic TME to deplete cholesterol and downregulate GLUT1 in xCT‐SLC7A11^high^ cancer cells, which in turn induced glucose and GSH depletion to lead to disulfidptosis of cancer cells. Elevated ROS levels from RT‐RDT and reduced GSH levels establish a positive feedback loop to induce immunogenic death of cancer cells. The mitochondria‐targeting ability of Th‐Ir‐DBB further enhances RT‐RDT‐mediated cancer immunotherapy due to the critical roles of mitochondria in cancer metabolism, radiation response, and redox balance. Cholesterol depletion also effectively downregulates multiple immune checkpoints in cancer cells [including PD‐L1 and cluster of differentiation 47 (CD47)] and T cells (including 2B4 and TIM‐3) to enhance the immune response of T cells in the TME. As a result, Th‐Ir‐DBB/dig plus low‐dose X‐ray irradiation effectively inhibited tumor growth in mouse models of colon and breast cancer.

## 2 Results and Discussion

### 2.1 Preparation and Characterization of Th‐Ir‐DBB/Dig

We targeted the synthesis of UiO frameworks because of their high chemical stability, structural predictability, and large pores.^[^ ^24^ ^]^ Positively charged Ir‐DBB was chosen as a bridging ligand due to its excellent photosensitizing property and ability to target mitochondria (Scheme S1, Figures S1 and S2, Supporting Information). Th_6_O_4_(OH)_4_ SBUs were chosen for the high Z number of Th (Z = 90) and the chemical similarity between Th^4+^ and Zr^4+^ ions. Th_6_‐Ir‐DBB was synthesized through a solvothermal reaction between Th_6_O_4_(OH)_4_(HCOO)_12_ clusters^[^ ^22^ ^]^ and Ir‐DBB in a mixture of N,N‐dimethylformamide, acetic acid, and water at 80 °C for 24 h (Figure 1a; Scheme S2, Supporting Information). Th‐Ir‐DBB displayed an octahedron‐like morphology with a diameter of 100 nm by transmission electron microscopy (TEM, Figure 1b) and a number‐averaged size of 106 nm by dynamic light scattering (DLS, Figure 1c). As the octahedral morphology is commonly observed for UiO‐type MOFs with hexa‐nuclear SBUs, the morphology of Th‐Ir‐DBB suggests the successful linking of Th_6_ clusters by Ir‐DBB ligands. Powder X‐ray diffraction (PXRD) patterns confirmed the adoption of the *fcu* topology in Th_6_‐Ir‐DBB, similar to that of UiO‐69 MOF (Figure 1d) with a 2θ value (3.8°) for the (111) reflection.^[^ ^25^ ^]^ X‐ray photoelectron spectroscopy (XPS) demonstrated the presence of both Th and Ir in Th‐Ir‐DBB (Figure 1e; Figure S3, Supporting Information). Inductively coupled plasma‐mass spectrometry (ICP‐MS), ultra–violet visible (UV–vis) spectroscopy (Figures S2 and S4, Supporting Information), and ^1^H NMR spectroscopy analyses (Figure S5, Supporting Information) of digested Th‐Ir‐DBB gave an empirical formula of Th_6_(µ_3_‐O)_4_(µ_3_‐OH)_4_(Ir‐DBB)_4.9_(HCOO)_0.6_(OH)_1.6_.

Th‐Ir‐DBB/Dig was prepared by mixing digitonin and Th‐Ir‐DBB in water at room temperature (Scheme S3, Supporting Information). Digitonin was chosen for its ability to selectively permeabilize plasma membranes by forming complexes with cholesterol and its widespread use in the study of membrane proteins, lipid rafts, and cholesterol‐dependent signaling pathways.^[^ ^26^ ^]^ Fourier‐transform infrared spectroscopy (FT‐IR) demonstrated successful loading of digitonin into Th‐Ir‐DBB/Dig with a broad ─OH stretching vibration band at 3394 cm^−1^ and a C─H stretching vibration band at 2927 cm^−1^ (Figure 1g; Figure S6, Supporting Information).^[^ ^27^ ^]^ The FT‐IR spectrum of Th‐Ir‐DBB/Dig also showed absorption peaks at 1452 and 1369 cm^−1^ for C─H deforming vibrations and at 1000–1200 cm^−1^ for C─O─H stretching vibration and C─O─C glycosidic vibration bands.^[^ ^28^ ^]^ Th‐Ir‐DBB/Dig showed a similar morphology and size to Th‐Ir‐DBB as demonstrated by TEM (Figure 1f; Figure S7, Supporting Information), scanning electron microscopy (SEM, Figure S8, Supporting Information) and DLS (Figure 1c). The ζ‐potentials of Th‐Ir‐DBB and Th‐Ir‐DBB/Dig were 39.2 ± 3.2 mV and 43.3 ± 1.9 mV (Figure 1h), respectively. The encapsulation efficiency of digitonin was determined to be 83.6%. Nitrogen adsorption measurements of Th‐Ir‐DBB/Dig showed type I isotherms with a Brunauer–Emmett–Teller (BET) surface area of 51 m^2^ g^−1^, which is slightly smaller than that of Th‐Ir‐DBB (62 m^2^ g^−1^, Figure S9, Supporting Information). This result is consistent with the successful loading of digitonin in Th‐Ir‐DBB. Th‐Ir‐DBB/Dig released 38.8% of digitonin under physiological conditions (pH 7.4) over 48 h but released 20.1% digitonin in 6 h and 84.8% digitonin in 48 h under an acidic condition (pH 5, Figure 1i; Figures S10 and S11, Supporting Information), likely due to weakened hydrogen bonding and electrostatic interactions between Th‐Ir‐DBB and digitonin under acidic conditions.^[^ ^29^ ^]^ X‐ray irradiation did not enhance the release of digitonin from Th‐Ir‐DBB/Dig (Figure S12, Supporting Information). PXRD studies confirmed that Th‐Ir‐DBB and Th‐Ir‐DBB/Dig retained crystallinity after incubation in PBS (1 mm) for 3 days or upon X‐ray irradiation (12 Gy), ensuring their stability for biological and radiotherapeutic applications (Figure 1j). TEM also demonstrated that the morphology of Th‐Ir‐DBB/Dig did not change after incubation in PBS solution (Figure S13, Supporting Information).

### 2.2 Radiotherapy‐Radiodynamic Therapy Performance

**Figure 2:**
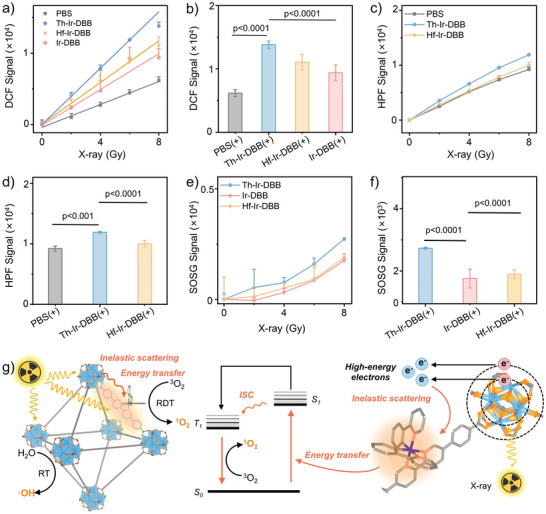
RT‐RDT performance. a,c,e) X‐ray dose‐dependent total ROS a), ·OH c), and ^1^O_2_ e) signals of PBS, Th‐Ir‐DBB, Hf‐Ir‐DBB, or Ir‐DBB measured by DCFH, HPF, or SOSG assays, respectively. b,d,f) Total ROS b), ·OH d), and ^1^O_2_ f) signals of PBS, Th‐Ir‐DBB, Hf‐Ir‐DBB, and Ir‐DBB, respectively, after 8 Gy X‐ray radiation. (a–f, *n* = 3). g) Proposed mechanism for the enhanced RT‐RDT effect of Th‐Ir‐DBB.

We evaluated the RT‐RDT performance of Th‐Ir‐DBB using various ROS probes. Hf‐Ir‐DBB was synthesized according to a previous report (Scheme S4, Supporting Information)^[^ ^30^ ^]^ and used as a comparator (Figure S14, Supporting Information). The total ROS generation at different doses of X‐rays was measured by 2′,7′‐dichlorodihydrofluorescein (DCFH) assay in test tubes (**Figure** **2**a,b).^[^ ^31^ ^]^ All groups showed linear increases with X‐ray doses, but Th‐Ir‐DBB exhibited a 2.24‐fold higher ROS generation than PBS at 8 Gy. In comparison, Hf‐Ir‐DBB, and Ir‐DBB only showed 1.79‐, and 1.52‐fold higher ROS generation than PBS, respectively. We next detected the generation of hydroxyl radicals (•OH) by hydroxyphenyl fluorescein (HPF) assay. Th‐Ir‐DBB showed a 1.19‐fold higher •OH signal than Hf‐Ir‐DBB (Figure 2c,d).^[^ ^32^ ^]^ The radiosensitization enhancement of Th‐Ir‐DBB primarily arises from the increased energy depositions on the heavier Th_6_ SBUs over the Hf_6_ SBUs in Hf‐Ir‐DBB. We also determined the generation of ^1^O_2_ via the RDT process using a singlet oxygen sensor green (SOSG). Th‐Ir‐DBB showed 1.55‐ and 1.44‐fold higher SOSG signals than Ir‐DBB ligand and Hf‐Ir‐DBB (Figure 2e,f), respectively. Thus, the enhanced ROS generation by Th‐Ir‐DBB is attributed to the potent radiosensitizing ability of Th and the strong RDT effect from Ir‐DBB.

As high‐Z elements in nMOFs serve as an effective antenna for X‐ray absorption, the presence of two high‐Z elements in Th‐Ir‐DBB further enhances the radiosensitization effect. Due to the more electropositive nature of Th over Hf, it is easier to eject the outer‐shell electrons from Th^IV^ in Th‐Ir‐DBB over Hf^IV^ in Hf‐Ir‐DBB upon X‐ray absorption through the photoelectric effect. Upon X‐ray irradiation, electron‐dense Th_6_ SBUs efficiently absorb X‐rays to generate ·OH and transfer energy to Ir‐DBB to generate ^1^O_2_. As a result, Th‐Ir‐DBB is expected to capture more photoelectrons and transfer energy to the Ir‐DBB ligands for a stronger RDT effect (Figure 2g).^[^ ^33^ ^]^

### 2.3 In Vitro Anticancer Mechanisms of Th‐Ir‐DBB/Dig

**Figure 3:**
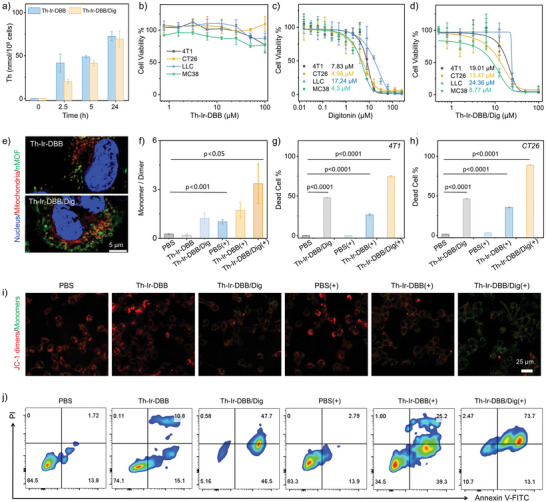
Uptake and cytotoxicity in cancer cells. a) Time‐dependent cellular uptake in 4T1 cells measured by ICP‐MS (*n* = 3). b–d) Viability of 4T1, CT26, LLC, and MC38 cells after incubation with digitonin b), Th‐Ir‐DBB (c, based on Th), or Th‐Ir‐DBB/Dig (d, based on digitonin) at different concentrations for 24 h. e) CLSM images of mitochondria in 4T1 cells treated with Th‐Ir‐DBB or Th‐Ir‐DBB/Dig. f) Relative signal intensity of JC‐1 monomers to dimers in 4T1 cells after different treatments (*n* = 3). g,h) Percentages of dead (late apoptotic and necrotic) 4T1 cells g) or CT26 cells h) after different treatments g,h, *n* = 3). i) CLSM images of JC‐1 stained 4T1 cells after different treatments. j) Apoptosis assay after staining with annexin V‐FITC/PI by flow cytometry.

To elucidate the anticancer mechanism of Th‐Ir‐DBB/Dig, we first determined the time‐dependent uptake of Th‐Ir‐DBB and Th‐Ir‐DBB/Dig by 4T1 murine breast cancer cells by ICP‐MS analysis (**Figure** **3**a). 4T1 cells rapidly uptook both Th‐Ir‐DBB and Th‐Ir‐DBB/Dig. We next evaluated the cytotoxicity of Th‐Ir‐DBB, digitonin, and Th‐Ir‐DBB/Dig in Lewis lung carcinoma (LLC), 4T1, murine colon adenocarcinoma (MC38), and murine colorectal carcinoma (CT26) cancer cells. Th‐Ir‐DBB exhibited minimal toxicity even at a concentration of 100 µm (based on Th) on LLC, 4T1, MC38, and CT26 cells (Figure 3b). Digitonin displayed IC_50_ values of 17.2, 5.0, 7.8, and 4.3 µm for LLC, CT26, 4T1, and MC38 cells, respectively. The digitonin IC_50_ values of Th‐Ir‐DBB/Dig increased to 24.4, 13.5, 19.0, and 8.8 µm for LLC, CT26, 4T1, and MC38 cells, respectively (Figure 3c,d). The increased digitonin IC_50_ values of Th‐Ir‐DBB/Dig over free digitonin supports the slow release of digitonin from Th‐Ir‐DBB/Dig in cell culture.

We next determined the mitochondria targeting ability of Hf‐Ir‐DBB and Th‐Ir‐DBB/Dig. Confocal laser scanning microscopy (CLSM) imaging showed mitochondria enrichment of Th‐Ir‐DBB and Th‐Ir‐DBB/Dig in 4T1 cells with strong co‐localization with mitochondria (Figure 3e; Figure S15, Supporting Information) but not lysosome (Figure S16, Supporting Information) as merged yellow areas. Next, the mitochondria were extracted and the mitochondria enrichment of Th‐Ir‐DBB and Th‐Ir‐DBB/Dig was quantified by ICP‐MS (Figure 3a; Figure S15c, Supporting Information). After incubation for 24 h, more than 43.5% and 38.7% of the internalized Th‐Ir‐DBB and Th‐Ir‐DBB/Dig, respectively, were localized in the mitochondria of 4T1 cells. TEM images of 4T1 cells treated with Th‐Ir‐DBB and Th‐Ir‐DBB/Dig further confirmed the mitochondria targeting effect (Figure S17, Supporting Information). CT26 cells also showed mitochondrial enrichment of Th‐Ir‐DBB and Th‐Ir‐DBB/Dig with strong co‐localization with mitochondria (Figure S18, Supporting Information). These results indicate that the incorporation of cationic Ir‐DBB ligands endows the nMOF with a large, dispersed positive charge for effective mitochondria targeting, and the loading of digitonin does not impact its mitochondria targeting property.

We subsequently measured the mitochondrial membrane potential (MMP) of 4T1 cells by 5,5′,6,6′‐tetrachloro‐1,1′,3,3′‐tetraethylbenzimidazolylcarbocyanine iodide (JC‐1) assay. Compared to PBS, Th‐Ir‐DBB plus X‐ray irradiation [denoted Th‐Ir‐DBB(+)] and Th‐Ir‐DBB/Dig(+) efficiently decreased the MMP with weaker red fluorescence of J‐aggregates and stronger green fluorescence of JC‐1 monomers. This result suggests that the synergistic effect between RT‐RDT and digitonin induces mitochondrial dysfunction via hypolethal oxidative stress (Figure 3f,i). Flow cytometry‐based apoptosis assay by double staining with Annexin V‐FITC and propidium iodide (PI) showed that the percentages of Annexin V^+^/PI^+^ cells increased from 0.2%, 1.2%, 2.3% and 1.3% for PBS, to 26.4%, 35.3%, 35.5% and 35.6% for Th‐Ir‐DBB(+), and to 74.5%, 88.8%, 51.6% and 97.8% for Th‐Ir‐DBB/Dig(+) for 4T1, CT26, LLC and MC38 cells, respectively. Th‐Ir‐DBB/Dig(+) increased the percentages of late apoptotic cancer cells compared to Th‐Ir‐DBB(+), suggesting that Th‐Ir‐DBB/Dig renders cancer cells vulnerable to RT‐RDT u(Figure 3g–j; Figure S23, Supporting Information).

### 2.4 Th‐Ir‐DBB/Dig Induces Disulfidptosis

**Figure 4:**
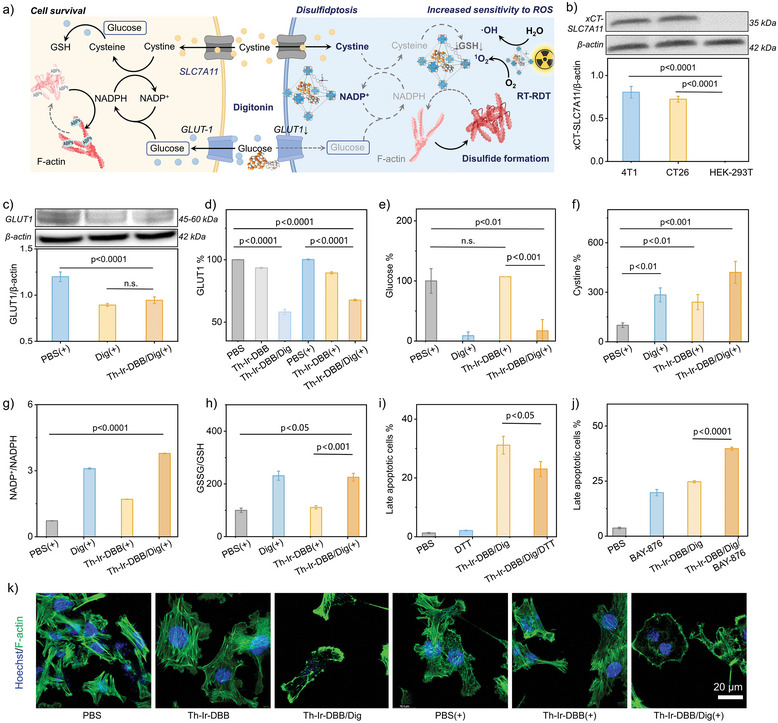
Disulfidptosis of cancer cells. a) Schematic showing the induction of disulfidptosis in xCT‐SLC7A11^high^ cancer cells. b) Western blot results and quantification of xCT‐SLC7A11 levels in 4T1, CT26 and HEK‐293T cells. c) Western blot results and quantification of GLUT1 levels in 4T1 cells after treatment with digitonin(+) or Th‐Ir‐DBB/Dig(+). d) Relative GLUT1 levels after different treatments as measured by flow cytometry (*n* = 3). e) Relative glucose levels in 4T1 cells after different treatments (*n* = 3). f) Relative cystine levels in 4T1 cells after different treatments. (*n* = 3) g) NADP^+^/NADPH ratios in 4T1 cells after different treatments. (*n* = 3) h) Relative GSSG/GSH ratios in 4T1 cells after different treatments (*n* = 3). i,j) Percentages of late apoptotic 4T1 cells after different treatments i,j, *n* = 3). k) F‐actin contraction during disulfidptosis.

We examined if digitonin could sensitize cancer cells to ROS generated by Th‐Ir‐DBB‐mediated RT‐RDT by altering cellular redox homeostasis (**Figure** **4**a). Western blot (WB) studies showed high expression of xCT‐SCL7A11 in 4T1, CT26, LLC, and MC38 cells, but not in human embryonic kidney 293 (HEK‐293T) normal cells (Figure 4b; Figure S19, Supporting Information). xCT‐SCL7A11 is crucial for maintaining redox balance and cell survival by mediating cystine uptake. As high levels of intracellular cystine are known to be toxic to cells due to its low solubility, cancer cells consume more NADPH to reduce cystine to cysteine. Consequently, xCT‐SLC7A11^high^ cancer cells are more susceptible to interventions that limit glucose or NADPH supply via disulfide accumulation.^[^ ^34^ ^]^

Digitonin permeabilizes cell membranes and depletes cholesterol, which affects lipid raft integrity and impacts the localization and function of GLUT‐1 in the membranes. However, the effect of digitonin on GLUT1 expression has not been established due to the involvement of multiple regulatory mechanisms, leading to a complex relationship between cholesterol depletion and GLUT‐1 expression. Considering the moderate expression of xCT‐SLC7A11 in 4T1 cells (Figure S19, Supporting Information) and their high invasiveness and tumorigenicity, which closely mimic human triple‐negative breast cancer in terms of growth and metastasis, we selected the 4T1 tumor model to investigate detailed mechanisms.^[^ ^35^ ^]^ We determined GLUT1 levels in 4T1 cells after digitonin or Th‐Ir‐DBB/Dig treatment by WB and flow cytometry (Figure 4c,d; Figure S20, Supporting Information). WB studies showed that digitonin and Th‐Ir‐DBB/Dig downregulated GLUT1 expression by 25.3% and 21.1%, respectively, which in turn decreased intracellular glucose levels by 87.1% and 60.3%, respectively (Figure 4e). Dig(+) and Th‐Ir‐DBB/Dig(+) further decreased intracellular glucose levels by 91.0% and 83.0%, respectively. Glucose depletion and ROS generation in 4T1 cells caused significant accumulation of intracellular cystine (Figure 4f), NADPH depletion (Figure 4g), and GSH depletion along with GSSG accumulation (Figure 4h). Dig(+) and Th‐Ir‐DBB/Dig(+) treatments increased the GSSG/GSH ratios by 2.31‐ and 2.25‐folds, respectively, over PBS(+) treatment.

To support these results, we investigated the impacts of *D*,*L*‐dithiothreitol (DTT) (Figure 4i; Figure S21, Supporting Information), a disulfide‐reducing agent, and BAY‐876 (Figure 4j; Figure S22, Supporting Information), a GLUT1 inhibitor, on the apoptosis of 4T1 cells. DTT reduced the late apoptotic 4T1 cells from 28.4% to 20.0%, suggesting Th‐Ir‐DBB/Dig induces apoptosis via disulfide formation. BAY‐876 plus Th‐Ir‐DBB/Dig increased late apoptotic 4T1 cells to 39.7% from 24.7% for Th‐Ir‐DBB/Dig alone, which suggests the contribution of glucose depletion to 4T1 cell death. Consistent with this hypothesis, Th‐Ir‐DBB/Dig‐mediated glucose depletion induced disulfide formation in actin cytoskeleton proteins, leading to morphological changes of 4T1 cells with F‐actin contraction and detachment from the plasma membrane (Figure 4k). The detachment of F‐actin from the inner cell membrane can have significant effects on cancer cells. F‐actin, a crucial component of the cell cytoskeleton, plays essential roles in maintaining cell shape, movement, and intracellular transport. F‐actin detachment on cancer cells might cause the loss of cell shape and integrity, impaired cell motility, disrupted intracellular transportation, altered signaling pathways, and response to therapies targeting the cytoskeleton or cell motility.^[^ ^36^ ^]^ We also measured cell death in LLC and MC38 cells following different treatments. Th‐Ir‐DBB/Dig and Th‐Ir‐DBB/Dig(+) treatments induced apoptotic cell death in an xCT‐SLC7A11‐dependent manner. MC38 cells high in xCT‐SLC7A11 showed increased susceptibility to killing by digitonin‐loaded MOF and enhanced synergy with X‐ray irradiation (Figure S23, Supporting Information). To gain insight into the therapeutic mechanism, we pre‐incubated 4T1 cells with the lipid peroxidation inhibitor ferrostatin for 12 h and then treated the cells with different treatments. Ferrostatin did not markedly inhibit cell death caused by Th‐Ir‐DBB/Dig and Th‐Ir‐DBB/Dig(+) (Figures S24 and S25, Supporting Information). This result supports the induction of disulfidptosis by Th‐Ir‐DBB/Dig and Th‐Ir‐DBB/Dig(+). Taken together, Th‐Ir‐DBB/Dig releases digitonin in xCT‐SLC7A11^high^ cancer cells to inhibit GLUT1 expression and reduce glucose uptake. Glucose depletion reduces NADPH levels, leading to intracellular accumulation of cystine and increasing the sensitivity of cancer cells to ROS generated from RT‐RDT.

### 2.5 Th‐Ir‐DBB/Dig(+) Induces Immunogenic Cell Death

**Figure 5:**
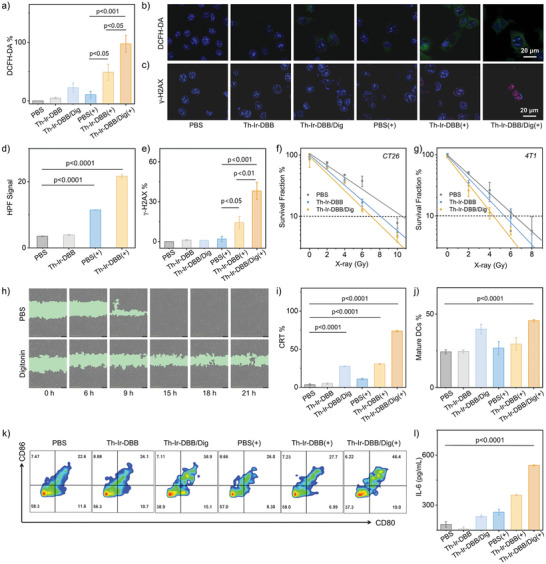
ICD of 4T1 cells. a,b) ROS signals in 4T1 cells after different treatments probed by DCFH‐DA via CLSM imaging. c) Expression levels of γ‐H2AX in 4T1 cells after different treatments were observed by CLSM. d) Relative HPF signal in 4T1 cells as measured by flow cytometry (*n* = 3). e) Relative expression of γ‐H2AX in 4T1 cells as measured by CLSM (*n* = 3). f,g) Linear dose‐fitting curves for surviving fractions (SF, log scale) of CT26 cells f) and 4T1 cells g) after X‐ray irradiation in clonogenic assays (*n* = 3). h) Wound closure of 4T1 cells after incubation with PBS or digitonin. i) Percentages of CRT‐positive 4T1 cells after different treatments as quantified by flow cytometry (*n* = 3). j,k) Percentages of mature DCs (CD80^+^CD86^+^CD11c^+^) detected by flow cytometry (*n* = 3). l) Secretion of IL‐6 in the supernatants of DCs after incubation with pretreated 4T1 cells.

We detected total ROS signals and ·OH generation in Th‐Ir‐DBB(+)‐treated 4T1 cells by DCFH and HPF assays, respectively. Th‐Ir‐DBB(+) gave 4.7‐fold and 1.9‐fold higher total ROS (**Figure** **5**a,b) and ·OH (Figure 5d; Figure S26, Supporting Information) signals than PBS(+), indicating strong Th‐Ir‐DBB‐mediated RT/RDT in cancer cells. We used phosphorylated H2AX (γ‐H2AX) assay to determine DNA double‐strand breaks (DSBs) in 4T1 cells by CLSM imaging, revealing that Th‐Ir‐DBB/Dig(+) and Th‐Ir‐DBB(+) showed 19.4 and 7.3 times higher γ‐H2AX expression than PBS(+) (Figure 5c,e; Figure S27, Supporting Information), respectively. Clonogenic assays, the gold standard for radiotoxicity assessment, were then performed to evaluate the radiosensitization effect of Th‐Ir‐DBB and Th‐Ir‐DBB/Dig on 4T1 cells. Upon X‐ray irradiation, Th‐Ir‐DBB and Th‐Ir‐DBB/Dig elicited dose enhancement of X‐rays, with dose modifying ratios at 10% (DMR_10%_) of 1.30 and 1.51, respectively, for CT26 cells, and 1.20 and 1.49, respectively, for 4T1 cells (Figure 5f,g; Figures S28 and S29, Supporting Information). These results indicate that Th‐Ir‐DBB/Dig‐mediated radiosensitization causes DNA damage to effectively inhibit colony formation. Additionally, we investigated the invasion and migration abilities of 4T1 cells by scratch wound analysis. While the PBS group showed wound closure in 15 h, digitonin treatment effectively inhibited cell migration likely by depleting cholesterol in cancer cell membranes (Figure 5h; Figure S30, Supporting Information).

Immunogenic cell death (ICD) represents a unique form of cell death that triggers the immune system to recognize and eliminate dying cells. Th‐Ir‐DBB/Dig(+) showed 6.8‐fold higher surface CRT expression (Figure 5i; Figure S31 and S32, Supporting Information), 4.1‐fold higher secretion of adenosine triphosphate (ATP, Figure S33, Supporting Information), and 14.9‐fold higher secretion of high mobility group box 1 (HMGB‐1, Figure S34, Supporting Information) than PBS(+). CRT acts as an “eat me” signal to promote phagocytosis of dying cells by antigen‐presenting cells. ATP release can also attract immune cells to the site of cell death, facilitating the clearance of dying cells and the initiation of immune responses. HMGB‐1 can facilitate antigen presentation by antigen‐presenting cells, such as DCs, thereby promoting adaptive immune responses against cancer cells.^[^ ^37^ ^]^ Th‐Ir‐DBB/Dig(+) showed 1.7‐fold higher percentage of mature DCs than PBS(+) (Figure 5j,k). Moreover, Th‐Ir‐DBB/Dig(+) secreted 2.1‐fold higher IL‐6, a pro‐inflammatory cytokine, than PBS(+) (Figure 5l). Thus, Th‐Ir‐DBB/Dig provides a novel bifunctional nanoparticle platform to induce ICD under X‐ray irradiation, which further stimulates the maturation of DCs to trigger an adaptive immune response against tumors.

### 2.6 Th‐Ir‐DBB/Dig Depletes Cholesterol to Downregulate Immune Checkpoints and Enhance Adaptive Immune Responses

**Figure 6:**
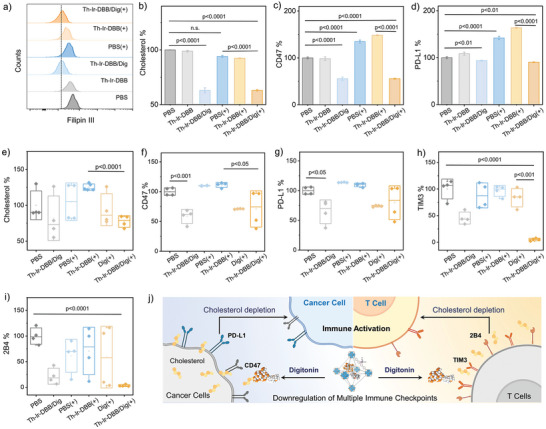
Cholesterol depletion and immune checkpoint downregulation. a,b) Relative cholesterol levels in 4T1 cells after different treatments quantified by flow cytometry (*n* = 3). c,d) Relative percentages of CD47 c) and PD‐L1 d) immune checkpoints in 4T1 cells quantified by flow cytometry (*n* = 3). e) Relative cholesterol levels in tumor tissue after different treatments. f,g) Relative expression of CD47 f) and PD‐L1 g) immune checkpoints in 4T1 tumor. h,i) Relative expression of TIM‐3 h) and 2B4 i) immune checkpoints in CD8^+^ T cells. j) Schematic showing the downregulation of multiple immune checkpoints in both cancer cells and T cells to enhance the overall immune response.

As RT can induce the secretion of immune suppressive cytokines and upregulate the expression of immune checkpoints in tumor tissue, it may hinder immune cell function in the TME and dampen the overall immune response.^[^ ^38^ ^]^ We anticipated that Th‐Ir‐DBB/Dig could consume cholesterol in cell membranes to enhance immune responses. We first used Filipin‐III, a cholesterol probe, to show that both Th‐Ir‐DBB/Dig and Th‐Ir‐DBB/Dig(+) depleted cellular cholesterol by 36.9% via flow cytometry analyses (**Figure** **6**a,b). We then examined the expression of CD47 and PD‐L1 immune checkpoints on 4T1 cell surfaces after different treatments. As expected, PBS(+) and Th‐Ir‐DBB(+) increased CD47‐positive 4T1 cells by 1.3‐folds and 1.5‐folds, respectively, and PD‐L1 positive 4T1 cells by 1.4‐folds and 1.6‐folds, respectively, over PBS treatment (Figure 6c,d; Figures S32, S35, and S36, Supporting Information). Slight increases of CD47 and PD‐L1 expressions following irradiation are related to DNA repair signaling as irradiated cancer cells utilize the Rad3‐related pathway for DNA double‐strand break repair, leading to CD47 and PD‐L1 upregulation.^[^ ^39^ ^]^ Interestingly, Th‐Ir‐DBB/Dig and Th‐Ir‐DBB/Dig(+) decreased CD47‐positive 4T1 cells by 1.8‐folds and PD‐L1‐positive 4T1 cells by 1.1‐folds over PBS treatment. Thus, Th‐Ir‐DBB/Dig effectively downregulates CD47 and PD‐L1 immune checkpoints on 4T1 cell surfaces.

To investigate if Th‐Ir‐DBB/Dig can alleviate T cell exhaustion in vivo, we determined the cholesterol levels in tumor tissues and CD47 and PD‐L1 expression levels in cancer cells as well as TIM‐3 and 2B4 levels in CD8^+^ T cells in 4T1 tumor‐bearing BALB/c mice. The mice with 4T1 tumors of ≈100 mm^3^ in sizes were randomized and treated with PBS, Th‐Ir‐DBB/Dig, PBS(+), Th‐Ir‐DBB(+), Dig(+), or Th‐Ir‐DBB/Dig(+). Th‐Ir‐DBB/Dig, digitonin, or Th‐Ir‐DBB were intratumorally injected at a Th dose of 0.5 µmol (equivalent Ir‐DBB dose of 0.4 µmol) and a digitonin dose of 0.19 µmol followed by 2 Gy/fraction X‐ray irradiation at the tumor site for three daily fractions (Figure S37, Supporting Information). On day ten post the last irradiation, Th‐Ir‐DBB/Dig, Dig(+), and Th‐Ir‐DBB/Dig(+) treatments lowered cholesterol levels in 4T1 cells by 1.3‐, 1.1‐, and 1.3‐folds, respectively, from PBS treatment (Figure 6e), leading to 1.7‐, 1.4‐, and 1.4‐fold reduction of CD47‐positive 4T1 cells (Figure 6f) and 1.6‐, 1.4‐, and 1.3‐fold reduction of PD‐L1 positive 4T1 cells (Figure 6g). The expression of TIM‐3 and 2B4 immune checkpoints in CD8^+^ T cells was also measured. Importantly, Th‐Ir‐DBB/Dig and Th‐Ir‐DBB/Dig(+) treatments significantly downregulated TIM‐3 expression by 54.5% and 94.9% (Figure 6h), respectively, and 2B4 expression by 78.4% and 96.6% (Figure 6i), respectively, from PBS treatment. These results show that Th‐Ir‐DBB/Dig effectively downregulates immune checkpoints in both cancer cells and T cells via cholesterol depletion (Figure 6j).

**Figure 7:**
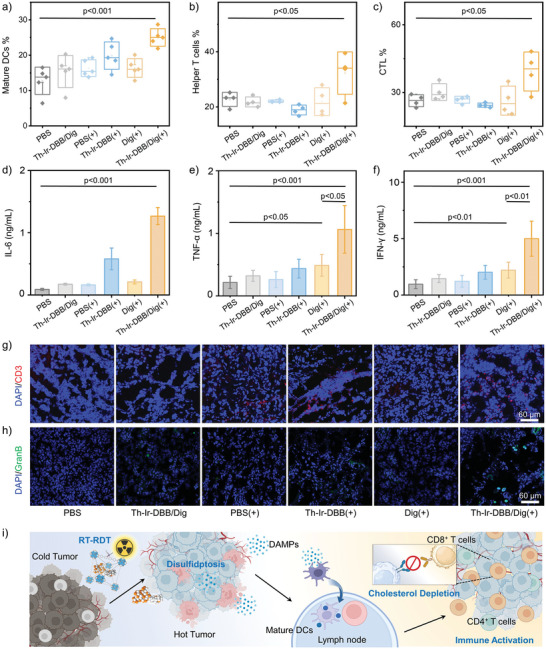
Stimulation of adaptive immune responses. a) Percentages of mature DCs (CD80^+^ CD86^+^) in CD11c^+^ cells. b) Percentages of helper T cells (CD3^+^CD4^+^) in CD45^+^ cells. c) Percentages of CTLs (CD3^+^CD8^+^) in CD45^+^ cells. d–f) Intratumoral concentrations of IL‐6 d), TNF‐α e), and IFN‐γ f) were detected by ELISA d–f, *n* = 4). g,h) CD3^+^ g) and GranB h) immunofluorescence staining of tumor tissue after different treatments. i) Schematic illustration of the immune response in tumors.

We next evaluated adaptive immune responses by examining immune cell activation and the release of inflammatory cytokines in 4T1 tumors. The infiltration of DCs and T cells in the TME was detected by flow cytometry. Th‐Ir‐DBB/Dig(+) treatment increased the percentage of mature DCs to 25.0% from 12.8% for PBS treatment (**Figure** **7**a; Figure S38, Supporting Information), suggesting the potential of mediating downstream immune responses through the proliferation of T cells.^[^ ^40^ ^]^ Flow cytometric analysis revealed that Th‐Ir‐DBB/Dig(+) treatment significantly increased the infiltration of CD3^+^CD4^+^ helper T cell percentage in CD45^+^ cells to 32.3% from 22.7% for PBS control (Figure 7b; Figure S39, Supporting Information). The other treatment groups had no effect on intratumoral T‐cell infiltration. Th‐Ir‐DBB/Dig(+) treatment increased CD3^+^CD8^+^ cytotoxic T cells (CTLs) percentage in CD45^+^ to 39.3% from 26.4% for PBS control while other treatment groups had no effect on the intratumoral infiltration of CTLs (Figure 7c; Figure S40, Supporting Information). Furthermore, the secretion of inflammatory cytokines in tumor tissues was quantified using ELISA. Th‐Ir‐DBB/Dig(+) treatment significantly increased the levels of pro‐inflammatory IL‐6 (Figure 7d), tumor necrosis factor‐alpha (TNF‐α, Figure 7e), and type‐II interferon‐gamma (IFN‐γ, Figure 7f). Immunofluorescence staining of tumor tissues supported the reinvigoration of T cells, with higher numbers of CD3^+^ T cells (Figure 7g) and high expression level of Granzyme B (GranB) in Th‐Ir‐DBB/Dig(+)‐treated tumor tissues (Figure 7h). These results indicate the generation of a more T‐cell‐inflamed TME by Th‐Ir‐DBB/Dig(+) treatment (Figure 7i).

### 2.7 In Vivo Suppression of Tumor Growth

**Figure 8:**
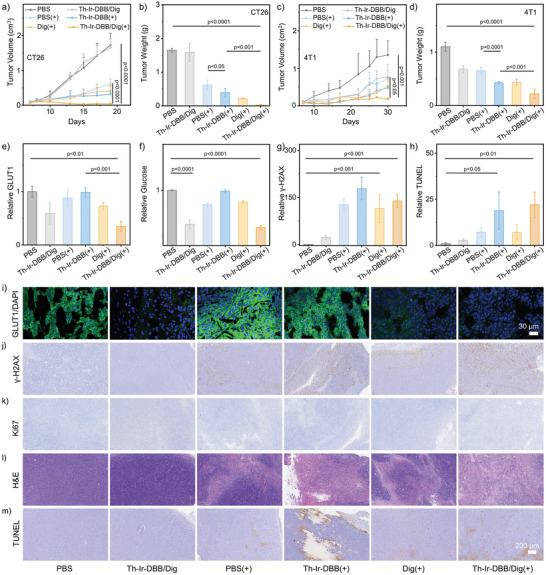
Inhibition of the subcutaneous CT26 and 4T1 tumors. a,b) Tumor volumes a) and tumor weights b) of subcutaneous CT26 tumors after different treatments. c,d) Tumor volumes c) and tumor weights d) of subcutaneous 4T1 tumors after different treatments. e,i) Expression levels of GLUT1 in 4T1 tumor tissue after different treatments. f) Relative glucose levels in 4T1 tumor tissue after different treatments. Expression levels of γ‐H2AX g,j) and Ki67 k) in 4T1 tumor tissues after different treatments. H&E l) and TUNEL h,m) staining of 4T1 tumors after different treatments.

The in vivo antitumor efficacy of Th‐Ir‐DBB/Dig was evaluated on subcutaneous CT26 murine colon carcinoma and 4T1 murine breast tumors of ≈80 and ≈90 mm^3^ in size, respectively. The mice were randomized and treated as shown in Figure S37 (Supporting Information). The tumors in PBS and Th‐Ir‐DBB/Dig groups exhibited rapid growth with similar trends, indicating minimal antitumor effect from Th‐Ir‐DBB/Dig alone (**Figure** **8**a–d). Irradiation of CT26 tumors and 4T1 tumors with 2 Gy/fraction of X‐rays for three daily fractions moderately inhibited tumor growth with tumor growth inhibition indices (TGIs) of 68.4% and 33.3%, respectively. Th‐Ir‐DBB(+) further inhibited tumor growth with TGIs of 85.4% and 56.8% for CT26 and 4T1 tumors, respectively. In contrast, Th‐Ir‐DBB/Dig(+) significantly inhibited the growth of both CT26 and 4T1 tumors with TGI values of 97.7% and 86.1%, respectively (Table S1, Supporting Information).

Immunofluorescence and immunohistochemistry staining analysis of 4T1 tumors 24 h post the last X‐ray irradiation revealed decreased GLUT1 expression, increased DSBs, reduced proliferation, and enhanced apoptosis of cancer cells when compared to the PBS group. Th‐Ir‐DBB/Dig, Dig(+), and Th‐Ir‐DBB/Dig(+) treatments decreased GLUT1 levels by 1.7‐, 1.4‐, and 2.9‐folds, respectively (Figure 8e,i). The decrease in GLUT1 expression was accompanied by a reduction in glucose levels in the tumor tissues (Figure 8f), leading to decreased nutrition supply and disulfidptosis in the tumors. Th‐Ir‐DBB/Dig(+) treatment showed 139‐fold higher DNA fragmentation as demonstrated by γ‐H2AX staining (Figure 8g,j) and 8.3‐fold lower Ki67‐positive cells (Figure 8k; Figure S41, Supporting Information) than PBS treatment. H&E staining of tumor tissues revealed sparser nuclei in the Th‐Ir‐DBB/Dig(+) group compared to Th‐Ir‐DBB(+) and Dig(+) groups (Figure 8l). Terminal deoxynucleotidyl transferase dUTP nick end labeling (TUNEL) immunostaining showed that Th‐Ir‐DBB(+)‐ and Th‐Ir‐DBB/Dig(+)‐treated tumors exhibited 18.9‐ and 22.1‐fold stronger tumor apoptosis, respectively, than PBS‐treated tumors (Figure 8h,m). These results show that Th‐Ir‐DBB/Dig(+) elicits strong antitumor effects via synergistic actions of Th‐Ir‐DBB‐mediated RT‐RDT and digitonin‐mediated disulfidptosis.

The biocompatibility of Th‐Ir‐DBB and Th‐Ir‐DBB/Dig was assessed through H&E staining of normal tissues and monitoring changes in the body weight of tumor‐bearing BABL/c mice. No abnormalities were detected in major organs such as the heart, liver, spleen, lungs, and kidneys (Figure S42, Supporting Information) nor was significant weight loss observed across all treatment groups (Figures S43 and S44, Supporting Information). Th‐Ir‐DBB and Th‐Ir‐DBB/Dig at a Th dose of 50 µm and a digitonin dose of 19.1 µm did not cause obvious hemolysis of red blood cells (Figure S45, Supporting Information). These findings suggest that Th‐Ir‐DBB/Dig serves as a biocompatible nanoplatform for cancer therapy.

## 3 Conclusion

In this work, we developed a novel mitochondria‐targeted and digitonin‐loaded nMOF to elicit potent antitumor effects with low‐dose X‐ray irradiation. Comprised of Th_6_O_4_(OH)_4_ SBUs and Ir(DBB)(ppy)^2+^ photosensitizing ligands, Th‐Ir‐DBB elicits strong RT‐RDT effects via potent radiosensitization for hydroxyl radical generation and efficient excitation of Ir‐DBB ligands for singlet oxygen production. Th‐Ir‐DBB/dig selectively releases digitonin in the acidic TME to deplete cholesterol and downregulate GLUT1 in xCT‐SLC7A11^high^ cancer cells for glucose and GSH depletion, leading to disulfidptosis of cancer cells by increasing their vulnerability to ROS. Additionally, digitonin‐mediated cholesterol depletion downregulates multiple immune checkpoints in both cancer cells and T cells to enhance the immune response to RT‐RDT. Consequently, Th‐Ir‐DBB/Dig effectively inhibits the growth of murine colon and breast tumors under low‐dose X‐ray irradiation. This study highlights the potential of utilizing the porous structures of nMOFs for the delivery of therapeutic molecules to overcome the physiological barriers of the TME and sensitize cancer cells to RT‐RDT.

## Conflict of Interest

The authors declare no conflict of interest.

## Supporting information



Supporting Information

## Data Availability

The data that support the findings of this study are available from the corresponding author upon reasonable request.
