# Supplementary material for: Digitonin‐Loaded Nanoscale Metal–Organic Framework for Mitochondria‐Targeted Radiotherapy‐Radiodynamic Therapy and Disulfidptosis
Source: Adv Mater. 2024 Sep 10;37(52):2405494. doi: 10.1002/adma.202405494 (PMC11891090; doi:10.1002/adma.202405494)
Supplement: Supplementary file 1 — Supporting Information [file ADMA-37-2405494-s001.pdf]

ADVANCED MATERIALS

Supporting Information

for *Adv. Mater.*, DOI 10.1002/adma.202405494

Digitonin-Loaded Nanoscale Metal–Organic Framework for Mitochondria-Targeted Radiotherapy-Radiodynamic Therapy and Disulfidptosis

*Wenyao Zhen, Yingjie Fan, Tomas Germanas, Langston Tillman, Jinhong Li, Abigail L. Blenko, Ralph R. Weichselbaum and Wenbin Lin**

WILEY-VCH

Supporting Information

Digitonin-Loaded Nanoscale Metal-Organic Framework for Mitochondria-Targeted Radiotherapy-Radiodynamic Therapy and Disulfidptosis

*Wenyao Zhen,[‡] Yingjie Fan,[‡] Tomas Germanas, Langston Tillman, Jinhong Li, Abigail L. Blenko, Ralph R. Weichselbaum, Wenbin Lin**

Materials

All starting chemicals were purchased from Sigma-Aldrich (USA) or ThermoFisher (USA) and used directly without purification. Filipin III, digitonin, *D*,*L*-dithiothreitol (DTT), BAY-876, anti-SLC7A11 antibody (produced in rabbit), NADP/NADPH quantitation kit and glutathione (GSH)/GSSG assay kit were purchased from Sigma-Aldrich (USA). Singlet oxygen sensor green (SOSG), ATP determination kit, anti-CD16/32, protein quantitative reagent kit-BCA, goat anti-rabbit lgG secondary antibody, anti-mouse lgG secondart antibody, glucose colorimetric detection kit, mitotracker red, lysosome tracker red, mitochondria isolation kit and collagenase I were purchased from Thermal Fisher Scientific (USA). Ferrostatin was purchased from TOCRIS Biotechne. Phosphate buffered saline (PBS) for cell culture, cell strainers (40 μm), and RPMI-1640 medium were purchased from Corning (USA). HMGB-1 ELISA kit was purchased from Chondrex, Inc. Trypsin-EDTA solution was purchased from Cytiva (USA). Actin-stain fluorescent phalloidins were purchased from Cytoskeleton, Inc. (USA). 2',7'-dichlorodihydrofluorescein diacetate (DCFH-DA), hydroxyphenyl fluorescein (HPF), dead cell apoptosis kit with annexin V Alexa Fluor 488 & propidium iodide (PI) were purchased from Invitrogen. Fetal bovine serum was purchased from VWR (USA). HyClone penicillin-streptomycin 100X solution was purchased from Cytiva (USA). Anti-CRT Alexa Fluor 488 was purchased from NOVUS Biologicals (USA). CellTiter 96® AQueous one solution cell proliferation assay (MTS) was purchased from Promega (USA). Beta-actin mouse mAb, phospho-histone H2A.X (Ser139) (γ-H2AX) rabbit monoclonal antibody, anti-rabbit IgG (H+L), F(ab')2 Fragment (Alexa Fluor® 488 Conjugate), GLUT1 (E4S6I) Rabbit mAb, calreticulin (D3E6) XP® Rabbit mAb, PD-L1 (D4H1Z) Rabbit mAb, and CD47 (E2V9V) Rabbit mAb were purchased from Cell Signaling Technology. APC anti-mouse CD86 antibody, PE-Cyanine 7 anti-mouse CD80 antibody, PE-Dazzle CD 11c antibody, granulocyte-macrophage colony stimulating factor (GM-CSF), and interleukin-4 (IL-4) were purchased from PeproTech. IL-6, IL-10, IFN-γ, and TNF-α ELISA kits were purchased from Invitrogen (USA). DNase I was purchased from Roche (USA). CD45-AlexaFluor488 (30-F11), CD11b-SuperBright600 (M1/70), F4/80-PerCP/Cy5.5 (BM8), CD11c-SuperBright436 (N418), CD3e-SuperBright600 (145-2C11), CD4-AlexaFluor488 (GK1.5), CD8a-APC-eFluor780 (53-6.7), CD244.2-PE (m2B4 (B6)458.1), TIM3-PE (RMT3-23). CD244.2-PE, CD47-PE, Gran B-FITC and PD-L1-APC were purchased from BioLegend. 4T1 cells were obtained from Prof. Stephen Kron at the University of Chicago. Human embryonic kidney (HEK) 293T cell line was purchased from American Type Culture Collection. CT26 cells were purchased from the American Type Culture Collection (ATCC, Virginia, USA).

Methods

Transmission electron microscopy (TEM) was carried out on an FEI Spirit 120 kV LaB6 Electron Microscope. Gel DOC^{TM} EZ imager (Bio-Rad) was used to capture Southern Blot images. BioTek Synergy HTX microplate reader was used to detect the absorption or FL intensity of 96-well plates. UV-Vis spectra were collected using a Shimadzu UV-2600 UV-Vis spectrophotometer. Dynamic light scattering (DLS) and ζ-potential measurements were performed on a Malvern Zetasizer Nano ZS instrument. Inductively coupled plasma-mass spectrometry (ICP-MS) data were collected using an Agilent 7700x ICP-MS and analyzed using an ICP-MS Mass Hunter version 4.6 C.01.06. Samples were diluted in a 2% HNO_3 matrix and analyzed with ^{159}Tb as internal standards against a 10-point standard curve between 1 ppb and 500 ppb ($R>0.999$ for ^{178}Hf). Data collection was performed in Spectrum Mode with triplicates per sample and 100 sweeps per replicate. Flow

cytometry data were collected on an LSR-Fortessa 4-15 HTS (BD Biosciences, USA) at the Cytometry and Antibody Technology Facility at the University of Chicago and analyzed by FlowJo software (Tree Star, USA). Nitrogen sorption experiments were performed with a Micromeritics 3Flex Adsorption Analyzer. Confocal laser scanning microscopy (CLSM) images were collected on a Leica Stellaris 8 laser scanning microscope at the Integrated Light Microscopy Facility at the University of Chicago, and analysis was done with ImageJ software (NIH, USA). Tissue sections were prepared with a ultramicrotome (Reichert/Leica Ultracut E, German). The histological slides were scanned on a CRi Pannoramic SCAN 40x whole slide scanner by Integrated Light Microscopy Core at the University of Chicago and analyzed with the QuPath-0.2.3 software. The wound healing experiment and cell population doubling (PD) time assessment were studied by IncuCyte S3 (Essen BioScience) at Cellular Screening Center at the University of Chicago. For X-ray irradiation in test tube and in vitro experiments, an RT250 orthovoltage X-ray machine model (Philips, USA) with fixed setting at 250 kVp, 15 mA and a built-in 1 mm Cu filter was used. The in vivo experiments were perforemd with X-RAD 225 with an X-ray dose rate of 0.04167 Gy/second. The X-ray dosimetry of both instruments was calibrated with an ionization chamber regularly by the Department of Radiation Oncology at the University of Chicago.

Synthesis of Ir-DBB

$[Ir(ppy)_2Cl]_2$ (1.07 g, 1.0 mmol), Me_2DBB (0.85 g, 2.0 mmol), methanol (25 mL), and chloroform (25 mL) were added to a 200 mL heavy-walled tube. The tube was degassed with N_2, sealed, and heated at 120 °C for 2 days. After cooling to ambient temperature, the solvents were removed under reduced pressure to afford pure Me_2DBB-Ir as an orange solid (1.92 g, 2.0 mmol, 100%). The solid was sonicated with ethanol and filtered to remove the remaining impurities.

Me_2DBB-Ir (0.96 g, 1.0 mmol), THF (50 mL), MeOH (50 mL), NaOH (1.5 g), and H_2O (20 mL) were added to a 250 mL flask. The solution was stirred under reflux overnight. After THF and MeOH were removed under reduced pressure, the solution was acidified by adding concentrated HCl until pH = 1 was reached and a yellow precipitate formed. The solid was collected by centrifugation, washed H_2O (40 mL) and MeOH (10 mL), and finally dried under vacuum to afford Ir-DBB in the diprotonated form (0.85 g, 0.91 mmol, 91%) as a fine yellow powder. ^1H NMR (500 MHz, DMSO-d_6): δ 9.05 (d, 2H), 8.66 (dd, 2H), 8.27 (d, 2H) 8.06 (d, 2H), 7.94 (m, 8H), 7.88 (d, 2H), 7.53 (d, 4H), 7.16 (t, 2H), 6.97 (t, 2H), 6.32 (d, 2H). HR-MS (ESI, positive mode) for $[IrC_{46}H_{32}N_4O_4H]^+$: m/z, calc'd 898.2131; found 898.2127.

Preparation of $Th_6O_4(OH)_4(CH_3COO)_{12}$ (Th_6) cluster

A white precipitate formed upon the addition of 1 mL of NH_4OH (30% w% aqueous solution) to a 1 mL solution of 0.5 M $Th(NO_3)_4$ in H_2O. The resulting white precipitate was washed several times with distilled water until the pH of the supernatant was near neutral. Next, 5 mL of 0.5 M acetic acid was added to the precipitate and the resulting mixture was centrifuged, and the supernatant was transferred to a glass vial. Evaporation of the solution yielded small colorless blocks of the Th_6 cluster.[1] The yield of Th_6 cluster was 89%.

Preparation of Th-Ir-DBB

Th-Ir-DBB was prepared from a solvothermal reaction between Th_6 cluster and Ir-DBB ($[Ir(H_2DBB)(ppy)_2]Cl$,[2] DBB = 4,4'-di(4-benzoato)-2,2'-bipyridine; ppy = 2-phenylpyridine). 1.2 mg Th_6 cluster, 1.6 mg Ir-DBB, 3 uL acetic acid, and 5 uL H_2O were mixed in 1 mL *N,N*-dimethylforamide (DMF) in a 1-dram vial. The mixture was heated in an oven at an 80 °C for 1

day to afford an orange precipitate, which was washed with DMF, and ethanol sequentially and dispersed in ethanol.

Hf-Ir-DBB were synthesized as described previously for comparison.[2] Typically, 0.5 mL of $HfCl_4$ solution (2.0 mg/mL in DMF), 0.5 mL of Ir-DBB solution (4.0 mg/mL in DMF), 1 μL of trifluoroacetic acid (TFA), and 5 μL of water were added to a 4 mL glass vial. The reaction mixture was kept in a 70 °C oven for 24 hours. The yellow precipitate was collected by centrifugation and washed with DMF and ethanol.

Synthesis of Th-Ir-DBB/Dig

200 μL of digitonin aqueous solution (10 mM) was dropwise added to 1 mL of Th-Ir-DBB aqueous dispersion (Th = 4.4 mM) under vigorous stirring at room temperature. Two hours later, the precipitates were collected by centrifugation at 14,000 g and washed with water 3 times. The supernatant was further treated with ultrafiltration (MWCO 10 kDa, Millipore) and the concentration of digitonin in the supernatant was then detected by UV-Vis to calculate the loading capacity of digitonin in Th-Ir-DBB/Dig. The digitonin loading capacity was calculated to be 38.1 mol% relative to Th-Ir-DBB based on Th (Th: Ir-DBB = 6 : 4.9). The loading (encapsulation) efficiency was 83.6%. SEM and TEM images of fresh prepared Th-Ir-DBB and Th-Ir-DBB/Dig were measured.

$$\text{Loading efficiency} = \text{n(loaded digitonin)} \div \text{n(added digitonin)} \times 100\%$$

$$\text{Loading capacity} = \text{n(loaded digitonin)} \div \text{n(Th - Ir - DBB/Dig, based on Th)} \times 100\%$$

For digitonin release assay, Th-Ir-DBB/Dig (Th: 5.24 mM, digitonin: 2 mM) was cultured in PBS (1 mM) at pH = 7.4, 6.5 or 5.0 at 37 ºC. At pre-set time intervals, the supernatant was obtained by ultrafiltration (MWCO 10 kDa, Millipore) and analyzed by UV-Vis. The release ratio with or without X-ray irradiation (6 Gy) was also measured according to the similar procedures as mentioned above.

Digestion of Th-Ir-DBB for UV-Vis measurements

50 μL Th-Ir-DBB dispersion, 900 μL DMSO, and 50 μL H_3PO_4 were mixed and sonicated for 1 hour, after which the mixture was diluted to a proper concentration for UV-Vis measurement. The absorption of Ir-DBB at 350 nm was used to calculate the concentration of Ir-DBB in Th-Ir-DBB after comparison with the standard curve of Ir-DBB in DMSO.

Digestion of Th-Ir-DBB for ^1H NMR analysis

Th-Ir-DBB was dried under vacuum overnight. 500 μL DMSO-d_6 and 50 μL D_3PO_4 were added to each solid. The mixture was sonicated for 1 hour, followed by the addition of 50 μL D_2O for ^1H NMR analysis.

Stability test of Th-Ir-DBB and Th-Ir-DBB/Dig

Th-Ir-DBB and Th-Ir-DBB/Dig were dispersed in 1 mL PBS (1 mM) with a Th concentration of 5 mM. 200 μL suspension was taken out after incubation for 3 days and centrifuged for PXRD measurement. For the stability test of Th-Ir-DBB and Th-Ir-DBB/Dig upon X-ray irradiation, Th-Ir-DBB and Th-Ir-DBB/Dig were dispersed in 1 mL H_2O separately with a Th concentration of 5 mM. 500 μL of each suspension was subjected to 12 Gy X-ray irradiation. PXRD measurements

were performed to evaluate the crystallinity of the irradiated nMOFs. The morphology of Th-Ir-DBB/Dig after incubation in PBS for 6 hours (1 mM, pH 7.4) was also measured by TEM.

ROS generation in test tubes

Total ROS was detected by the DCFH assay. 0.5 mL DCFH-DA (1 mM) in DMSO was hydrolyzed with 2 mL NaOH (10 mM) aqueous solution in the dark for 30 minutes under vigorous stirring, and the hydrolysis process was stopped by adding 10 mL PBS (25 mM, pH 7.4) to afford DCFH stock as the detection probe of total ROS in test tubes. PBS, Hf-Ir-DBB (Hf: 5 μM), Ir-DBB (4 μM), or Th-Ir-DBB (Th: 5 μM) in PBS solution (pH 7.4, 10 mM) was added to the freshly prepared DCFH solution with a final DCF concentration of 5 μM. The concentration of Ir-DBB was selected based on its ratio to Th in the nMOF (Th: Ir-DBB = 6 : 4.9). The PBS solution with the same DCFH concentration served as a blank control. 200 μL of each suspension was added to 96-well plates (n = 3), and the whole plate was irradiated with X-ray at different doses (0, 2, 4, 6, and 8 Gy). The fluorescence signal (E_x. 485/20 nm; E_m. 520/20 nm) was collected with an HTX microplate reader immediately after X-ray irradiation.

The generation of $^1O_2$ was detected by SOSG assay. Hf-Ir-DBB (Hf: 5 μM), Th-Ir-DBB (Th: 5 μM), Ir-DBB (3.75 μM) in PBS solution (pH 7.4, 10 mM) was added to the freshly prepared SOSG solution with a final SOSG concentration of 5 μM. The PBS solution with the same SOSG concentration served as a blank control. 200 μL of each suspension was added to 96-well plates (n = 3), and the whole plate was irradiated by X-ray for different dose (0, 2, 4, 6, and 8 Gy). The fluorescence signal (E_x. 485/20 nm; E_m. 520/20 nm) was collected with an HTX microplate reader immediately after X-ray irradiation.

The generation of ·OH was detected by HPF assay. Hf-Ir-DBB (Hf: 5 μM), Th-Ir-DBB (Th: 5 μM) in PBS solution (pH 7.4, 10 mM) was added to the freshly prepared HPF solution with a final HPF concentration of 5 μM. The PBS solution with the same HPF concentration served as a blank control. 200 μL of each suspension was added to 96-well plates (n = 3), and the whole plate was irradiated by X-ray for different dose (0, 2, 4, 6, 8 Gy). The fluorescence signal (E_x. 485/20 nm; E_m. 520/20 nm) was collected with an HTX microplate reader immediately after X-ray irradiation.

Cell culture

4T1 and CT26 cells were cultured in RPMI-1640 medium supplemented with 10 % fetal bovine serum (filtered), 1 % HyClone penicillin-streptomycin 100× solution, and cultured in a humidified atmosphere containing 5 % CO_2 at 37 °C. HEK-293T, Lewis lung carcinoma (LLC) and MC38 murine colon cells were cultured in Dulbecco's Modified Eagle's Medium (DMEM) supplemented with 10 % fetal bovine serum (filtered), 1 % HyClone penicillin-streptomycin 100× solution, and cultured in a humidified atmosphere containing 5 % CO_2 at 37 °C. Mycoplasma was tested on a regular basis by MycoAlert detection kit.

Cellular uptake

The cellular uptake of Th-Ir-DBB and Th-Ir-DBB/Dig was evaluated on 4T1 cells. The cells were seeded in 6-well plates at a density of 5 $\times 10^5$/well and cultured overnight. Th-Ir-DBB or Th-Ir-DBB/Dig was added at an equivalent Th concentration of 50 μM into medium (n = 3). The cells were incubated in a 37 °C incubator for 0, 2.5, 5, and 24 hours. At each time point, the medium was aspirated, the cells were washed with PBS three times, trypsinized, collected by centrifugation, and counted with a hemocytometer. The cell pellets were digested with a mixture of 99% (v/v) concentrated nitric acid and 1% (v/v) hydrofluoric acid for 48 hours and the Th concentration was

determined by ICP-MS. The mitochondria were isolated by the mitochondria isolation kit according to the manufacturer's instructions, and the Th concentration in mitochondria was measured by ICP-MS.

Colocalization study by CLSM

4T1 or CT26 cells were seeded in the cell culture dishes (1.5×10^5 cells per dish) and cultured overnight. The cells were washed with PBS twice and stained with 500 nM mitotracker red probe (1 mL) or lysosome tracker red probe (1 mL, 500 nM) at 37 ºC for 20 minutes. Then, Th-Ir-DBB or Th-Ir-DBB/Dig was added at an equivalent Th concentration of 80 μM in the medium and incubated for 6 hours. The cells were washed with PBS three times, and observed on a Leica Stellaris 8 microscope.

In vitro cytotoxicity

The cytotoxicity of digitonin, Th-Ir-DBB, or Th-Ir-DBB/Dig on CT26, or 4T1 cells was detected by MTS assay. 100 μL of 4T1, CT26, LLC or MC38 cells (5.0×10^3 cells/well) were seeded in 96 well plates, 24 h later, RPMI-1640 medium containing different concentrations of digitonin, Th-Ir-DBB, or Th-Ir-DBB/Dig were added and the cells were incubated for another 24 hours. After that, 100 μL of 10 % (v/v) of MTS reagent was added to each well. 60-120 minutes later, the absorbance of each well at 490 nm was read by a Synergy HTX plate reader to calculate cell viability.

Apoptotic cell death

To quantify the apoptotic status of cells, 4T1, LLC, MC38 or CT26 cells were seeded in 6-well plates at a density of 1.5×10^5/well and cultured overnight. The cells were added with PBS, Th-Ir-DBB, or Th-Ir-DBB/Dig at an equivalent Th concentration of 50 μM and digitonin concentration of 19.1 μM. The cells were cultured for another 12 hours, and irradiated with X-ray (6 Gy). 12 hours later, the cells were washed with PBS, trypsinized to afford single cell suspensions. The cells were stained with the dead cell apoptosis kit with Annexin-V Alexa Fluor 488 & PI following the manufacturer's protocol and resuspended in the binding buffer for flow cytometric analysis (Annexin-V in FITC channel, PI in PE-dazzle 594 channel). Apoptosis of 4T1 cells pre-treated with reducing agents that prevent disulfide stress (DTT, 4 mM, 30 min) or GLUT1 inhibitor BAY-876 (5 μM, 2 h) was also measured as above. Apoptosis of 4T1 cells pre-incubated with ferrostatin (20 μM, 12 h) was also measured as above.

Cholesterol depletion

4T1 cells were seeded in 6-well plates at a density of 2×10^5/well and cultured overnight. The cells were added with PBS, Th-Ir-DBB, or Th-Ir-DBB/Dig at an equivalent Th concentration of 50 μM and digitonin concentration of 19.1 μM. The cells were cultured for another 24 hours, and irradiated with X-ray (6 Gy). 12 hours later, the cells were washed with PBS, trypsinized to afford single cell suspensions, and collected by centrifugation (300 *g*, 4 min). The cells were fixed with 4% paraformaldehyde at RT for 20 minutes. The cells were washed with PBS 3 times and stained with Filipin III PBS solution (0.1 mg/mL) at 4 ºC for 30 min. Then, the cells were washed with FACS buffer 3 times, and the signal of Filipin III was detected by flow cytometry (BV421 channel).

CRT expression

The expression level of CRT was also evaluated by flow cytometry. 4T1 cells were seeded in 6-well plates at a density of 2×10^5/well and cultured overnight. The cells were added with PBS,

Th-Ir-DBB, or Th-Ir-DBB/Dig at an equivalent Th concentration of 100 μM and digitonin concentration of 38.16 μM and cultured for another 6 hours, and irradiated with X-ray (6 Gy). 24 hours later, the cells were washed with PBS, trypsinized to afford single cell suspensions, and collected by centrifugation (300 g, 4 min). The cells were stained with anti-CRT Alexa Fluor 488 (1 : 150 dilution in 0.5 % BSA PBS solution) on ice for 30 minutes, washed with PBS once and resuspended in 0.5 % BSA PBS solution for flow cytometry analysis (FITC channel).

ATP and HMGB-1 secretion

4T1 cells were seeded in 6-well plates at a density of 2×10^5/well and cultured overnight. The cells were added with 1 mL of PBS, Th-Ir-DBB, or Th-Ir-DBB/Dig at an equivalent Th concentration of 50 μM and digitonin concentration of 19.08 μM and cultured for 12 additional hours, and irradiated with X-ray (6 Gy). 24 hours later, the supernatant of cell culture medium was collected and the cells were removed by centrifugation (300 g, 4 min). The ATP and HMGB-1 contents in the supernatants were measured using the ATP determination kit and HMGB-1 ELISA kit, respectively, following the manufacturers' instructions.

DNA damage

For CLSM imaging, CT26 cells were seeded in cell culture dishes at a density of 1.5×10^5 and cultured overnight. The cells were added with PBS, Th-Ir-DBB, or Th-Ir-DBB/Dig at an equivalent Th concentration of 50 μM and digitonin concentration of 19.1 μM. The cells were cultured for another 24 hours, and irradiated with X-ray (6 Gy). 12 hours later, the cells were washed with PBS and fixed with 4% paraformaldehyde at RT for 20 minutes. The cells were again rinsed with PBS, blocked and permeabilized with 5% FBS + 0.3% Triton-X in PBS at RT for 1 hour. After blocking, cells were incubated with the γ-H2AX primary antibody (1:500) in 1% BSA + 0.3% Triton-X in PBS at RT for 1 hour. The cells were then washed with PBS and incubated with the Alexa Fluor 488 conjugated secondary antibody (1:3000) in 1% BSA + 0.3% Triton-X in PBS at RT for 1 hour. Afterwards, the cells were washed with PBS and further incubated with Hoechst 33342 (10 μg mL^{-1}) in PBS for 10 minutes at room temperature to visualize cell nuclei. Finally, the cells were washed with PBS 3 times and observed on a Leica Stellaris 8 confocal microscope.

Intracellular generation of ROS

The in vitro generation of ROS upon X-ray irradiation was detected by CLSM with the DCFH-DA probe. 4T1 cells were seeded in cell culture dishes at a density of 1.5×10^5 and cultured overnight. The cells were stained by DCFH-DA medium solution (30 μM) for 30 min at 37 ºC. After that, the cells were washed by PBS for 3 times and added with PBS, Th-Ir-DBB, or Th-Ir-DBB/Dig at an equivalent Th concentration of 50 μM and digitonin concentration of 19.1 μM. The cells were cultured for another 24 hours and irradiated with X-ray (6 Gy), and then cultured for another 12 h. The medium was aspirated. The cells were rinsed with PBS three times and observed on a Leica Stellaris 8 confocal microscope.

Intracellular generation of ·OH

The generation of ROS upon X-ray irradiation was detected by CLSM with the HPF probe. 4T1 cells were seeded in cell culture dishes at a density of 1.5×10^5 and cultured overnight. The cells were stained by HPF medium solution (10 μM) for 30 min at 37 ºC. After that, the cells were washed with PBS for 3 times and added PBS, Th-Ir-DBB, or Th-Ir-DBB/Dig at an equivalent Th concentration of 50 μM and digitonin concentration of 19.1 μM. The cells were cultured for

another 24 hours and irradiated with X-ray (6 Gy), and then cultured for another 12 h. The medium was aspirated. The cells were rinsed with PBS three times and observed on a Leica Stellaris 8 confocal microscope.

Western blot analysis

4T1 cells were seeded in 6-well plates (2×10^5 cells/well) and cultured for 24 h. After that, the cells were washed with PBS 3 times and added PBS, Th-Ir-DBB, or Th-Ir-DBB/Dig at an equivalent Th concentration of 50 μM and digitonin concentration of 19.1 μM. The wells were cultured for another 24 hours and irradiated with X-ray (6 Gy), and then cultured for another 12 h. The medium was aspirated, the cells were washed with cold PBS twice and subjected to RIPA lysis buffer on ice. After grinding, the cell lysates were collected and centrifuged (14000 ×*g*, 15 min) at 4°C. The proteins in the supernatants were separated on SDS-PAGE and transferred to a PVDF membrane. The membrane was blocked with TBST buffer containing 5% non-fat powdered milk for 1 h. Thereafter, the membrane was incubated with diluted primary antibodies to CRT (1 : 1000), CD47 (1 : 1000), PD-L1 (1 : 1000), at 4°C overnight. After extensive washing with TBST buffer, the membrane was incubated with horseradish peroxidase-coupled secondary antibody (goat anti-rabbit IgG) for 2 h at room temperature. After washing away the secondary antibodies, the membrane was incubated with Pierce ECL Plus Western Blotting Substrate (Thermo Fisher Scientific, USA) and visualized using a FluorChem R system (ProteinSimple, USA).

The expression of GLUT1 after PBS(+), Dig(+) or Th-Ir-DBB/Dig(+) treatment was also measured according to the procedures described above with diluted primary antibodies GLUT1 (1 : 1000) at 4°C overnight

The expression of xCT-SLC7A11 in 4T1, CT26, HEK-293T, LLC, and MC38 cells was measured using the procedures described above. 5×10^6 cells were washed with cold PBS twice and subjected to RIPA lysis buffer on ice. After grinding, the cell lysates were collected and centrifuged (14000 ×*g*, 15 min) at 4°C. The proteins in the supernatants were separated on SDS-PAGE and transferred to a PVDF membrane. The membrane was blocked with TBST buffer containing 5% non-fat powdered milk for 1 h. Thereafter, the membrane was incubated with diluted primary antibodies to xCT-SLC7A11 (1 : 1000) at 4°C overnight. The remaining steps are similar to those described above.

Cytoskeletal protein observation

Acti-stain fluorescent phalloidins were used to stain the F-actin in fixed cells. 4T1 cells were seeded in cell culture dishes at a density of 1.5×10^5 and cultured overnight. The cells were added PBS, Th-Ir-DBB, or Th-Ir-DBB/Dig at an equivalent Th concentration of 50 μM and digitonin concentration of 19.1 μM. The cells were cultured for another 12 hours, and irradiated with X-ray (6 Gy). 12 hours later, the cells were washed with PBS and fixed with 4% paraformaldehyde at room temperature for 20 minutes. Next, the cells were permeabilized in a permeabilization buffer at room temperature for 5 min. The cells were then washed once with PBS at room temperature for 30 s and 200 μl of 100 nM Acti-stain 488 phalloidin PBS solution was added to the cells followed by incubation at room temperature for 30 min. The cell nuclei were stained by Hoechst (10 μg mL^{-1}) at room temperature for 10 min, and the calcium ion contents in the cells were observed by CLSM (E_x: 488 nm).

Detection of intracellular GSH and GSSG

GSH/GSSG assay kit was used to evaluate the intracellular GSSG/GSH ratio through a colorimetric method. 4T1 cells were seeded in 6-well plates (2×10^5 cells/well) and cultured for 24 h. After that, the cells were washed with PBS 3 times and added PBS, digitonin, Th-Ir-DBB, or Th-Ir-DBB/Dig at an equivalent Th concentration of 50 μM and digitonin concentration of 19.1 μM. The wells were cultured for another 6 hours and irradiated with X-ray (6 Gy), and then cultured for another 24 hours. The medium was aspirated, and the cells were washed with PBS, trypsinized to afford single cell suspensions, and collected by centrifugation (300 g, 4 min). The intracellular contents of GSH and GSSG were measured according to the manufacturer'ss instructions.

Detection of intracellular $NADP^+$ and NADPH

NADP/NADPH quantitation kit was used to evaluate the intracellular $NADP^+$/NADPH ratio through a colorimetric method. 4T1 cells were seeded in 6-well plates (2×10^5 cells/well) and cultured for 24 h. After that, the cells were washed with PBS 3 times and added PBS, digitonin, Th-Ir-DBB, or Th-Ir-DBB/Dig at an equivalent Th concentration of 50 μM and digitonin concentration of 19.1 μM. The wells were cultured for another 6 hours and irradiated with X-ray (6 Gy), and then cultured for another 24 hours. The medium was aspirated, and the cells were washed with PBS, trypsinized to afford single cell suspensions, and collected by centrifugation (300 g, 4 min). The intracellular $NADP^+$/NADPH ratio was measured according to the manufacturer's instructions.

Detection of intracellular glucose

Glucose assay kit was used to evaluate the intracellular glucose through colorimetric method. 4T1 cells were seeded in 6-well plates (2×10^5 cells/well) and cultured for 24 h. After that, the cells were washed with PBS 3 times and added PBS, digitonin, Th-Ir-DBB, or Th-Ir-DBB/Dig at an equivalent Th concentration of 50 μM and digitonin concentration of 19.1 μM. The wells were cultured for another 6 hours and irradiated with X-ray (6 Gy), and then cultured for another 24 hours. The medium was aspirated, cells were washed with PBS, trypsinized to afford single cell suspensions, and collected by centrifugation (300 g, 4 min). The intracellular content of glucose was measured according to the manufacturer' s standard instructions.

Detection of intracellular cystine

Cystine assay kit was used to evaluate the intracellular cystine through measuring the fluorescence). 4T1 cells were seeded in 6-well plates (2×10^5 cells/well) and cultured for 24 h. After that, the cells were washed with PBS 3 times and added PBS, digitonin, Th-Ir-DBB, or Th-Ir-DBB/Dig at an equivalent Th concentration of 50 μM and digitonin concentration of 19.1 μM. The wells were cultured for another 6 hours and irradiated with X-ray (6 Gy), and then cultured for another 24 hours. The medium was aspirated, cells were washed with PBS, trypsinized to afford single cell suspensions, and collected by centrifugation (300 g, 4 min). The intracellular content of cystine was measured according to the manufacturer' s standard instructions. The fluorescence signal (E_x. 485/20 nm; E_m. 520/20 nm) was collected with an HTX microplate reader after being incubated with working solution for 30 min at 37°C.

Wound healing

The wound healing assay was performed to evaluate the invasion and migration ability of 4T1 cells. The cells were first seeded in an Incucyte Imagelock 96-well plate (Sartorius) at a density of 5×10^5 cells/mL with 100 μL medium per well and cultured overnight. The wound was first created

with an Incucyte 96-well wound maker tool. The cells were washed with PBS twice, and PBS or digitonin at different concentrations in medium was added to the wells. The cells were incubated for 4 additional hours (n = 3). Then, the cells were put into IncuCyte S3 for live imaging for up to 24 hours and analyzed with a scratch wound analysis module.

Clonogenic assay

CT26 cells and 4T1 cells were seeded in 6-well plates at a density of 1.5×10^5 cells/well and cultured overnight. The cells were incubated with PBS, Th-Ir-DBB, or Th-Ir-DBB/Dig at an equivalent Th concentration of 40 μM for 4 hours, and then irradiated with X-ray at different doses (n = 3). The cells were washed with PBS twice and then trypsinized to afford single cell suspensions. The cells were counted and diluted, then 200 cells were seeded in each well of 6-well plates and cultured in 2 mL medium for 7 more days. When an appropriate colony size was observed, the plates were rinsed once with PBS, fixed with 4% paraformaldehyde for 20 minutes at at room temperature, and washed with PBS once. The 6-well plates were then scanned and analyzed with IncuCyte S3 (Essen BioScience) at Cellular Screening Center at the University of Chicago in the whole well mode with a 4× objective. The colonies were identified with IncuCyte 2021A software in a cellular resolution and the confluence was used as a parameter to calculate the plating efficiency (PE) and surviving fraction (SF):

$$PE = \frac{Confluence\ (0\ Gy, PBS)}{Cell\#\ (0\ Gy, PBS)} \qquad SF(D, MOF) = \frac{Confluence\ (D, MOF)}{Cell\#\ (D, MOF) \times PE}$$

Where D was the radiation dose and Cell# (D, MOF) was the number of cells seeded for a certain radiation dose D and a certain treatment group.

The dose modifying ratio at a 10% ($DMR_{10\%}$) was used as a parameter to assess radiosensitization effect and defined as the ratio of doses under reference conditions to produce a 10% SF:

$$DMR_{10\%} = \frac{D_{PBS}}{D_{MOF}}$$

In vitro stimulation of dendritic cells (DCs)

6 week-old female C57BL/c mice were used to isolate bone marrow derived immature DCs. The bone marrow was obtained by flushing the femur and tibia with PBS. After lysis of red blood cells, the cells were seeded in a cell culture dishes in RPMI 1640 containing additional 20 ng/mL of granulocyte-macrophage colony stimulating factor (GM-CSF) and 10 ng/mL of interleukin-4 (IL-4). Fresh medium was added to the culture dishes every two days. On the 6th day, the non-adherent and loosely adherent cells were collected. 3×10^6 non-adherent and loosely adherent cells in 1 mL medium were added in 6-well and 0.5 mL of 4T1 cells pretreated with PBS, Th-Ir-DBB, Th-Ir-DBB/Dig, PBS(+), Th-Ir-DBB(+), Th-Ir-DBB/Dig(+) at a Th concentration of 25 μM and digitonin concentration of 9.5 μM for 24 h. Then, DCs were obtained by centrifugation at 300 ×g and the supernatants were collected for cytokine analysis by IL-6 assays. The collected cells were further blocked with anti-CD16/32 antibodies (1 : 100) at 4 °C for 20 min, and stained with APC anti-mouse CD86 antibody (1 : 100), PE-Cyanine 7 anti-mouse CD80 Antibody (1 : 50) and PE-Dazzle CD 11c (1 : 100) in 100 μL PBS containing 1 % BSA for 30 min at at room temperature. The cells were rinsed with PBS containing 0.5 % BSA twice and evaluated by flow cytometry. The secretion of IL-6 in the supernatant of DCs was measured by ELISA kit following the manufacturer's instructions.

Anti-tumor efficacy on CT26 and 4T1 tumor models

BALB/c mice (6-8 weeks) were obtained from Charles River Laboratories, Inc (USA) and bred in house at the animal facility at the University of Chicago. The study protocol was reviewed and approved by the Institutional Animal Care and Use Committee (IACUC) at the University of Chicago. CT26 or 4T1 tumors were established on BALB/c mice by inoculating 2 × 10^6 cells/mouse subcutaneously onto the right flanks at day 0. When the tumor volumes reached ~80 or ~90 mm^3 for CT26 and 4T1 model, respectively, the mice were randomized. Th-Ir-DBB, digitonin, or Th-Ir-DBB/Dig was intratumorally injected at an equivalent Th dose of 0.5 μmol and digitonin dose of 0.19 umol in 20 μL PBS (n = 5). 6-8 hours later, the mice were anaesthetized with 2.5% (v/v) isoflurane/O_2 and mounted onto the X-Rad 225 irradiator. The tumors were irradiated with 2 Gy X-ray/fraction for 3 consecutive days. The lengths and widths of tumors were measured with an electronic caliper (tumor volume = length × $width^2/2$) and body weights were monitored with an electronic scale. At the endpoint of the experiments, the mice were euthanized, and the tumors and major organs were sectioned for hematoxylin-eosin (H&E) staining to evaluate general toxicity. The tumor growth inhibition index (TGI) was defined as the equation below:

$$TGI = \left(1 - \frac{\frac{T_e}{T_s} \Big/ \frac{C_e}{C_s}}{1 - \frac{C_s}{C_e}}\right) \times 100\%$$

Where T_e, T_s, C_e, and represent average tumor volumes of treated mice at endpoint, treated mice at starting-point, control mice at endpoint and control mice at starting-point, respectively.

Immune response evaluation

Subcutaneous 4T1 tumor-bearing BALB/c mice (n = 5) were treated with PBS, Th-Ir-DBB/Dig, PBS(+), Th-Ir-DBB+), Dig(+), or Th-Ir-DBB/Dig(+) as described above. The tumors were harvested at day 14 for immune cell profiling by flow cytometry. The tumors were digested with 600 μL of RPMI-1640 + 0.5 mg/mL collagenase I + 50 μg/mL DNase I cocktail at 37 °C for 45 minutes. The digests were neutralized with 6 mL RPMI-1640 medium with 10 % FBS and gently ground and filtered through sterile cell strainers (40 μm, Corning) to obtain single cell suspensions (~10^7 cells/mL). The supernatants were collected by centrifugation in 15 mL ep tubes at 300 ×g for 10 minutes at 4 °C for cytokine analysis by IL-6, TNF-α and IFN-γ assay kits, and the concentration of IL-6 or TNF-α in the tumor tissue normalized based on the tumor mass. While the cell pellets were collected, and the red blood cells were lysed with ACK buffer (ThermoFisher Scientific, 2 mL per sample), and the remaining cells were washed by FACS buffer (0.5 % BSA in PBS), transferred to a round-bottom 96-well plate, and stained first with Fixable Viability Dye eFluor 506 (ThermoFisher Scientific, 1 : 1000 in FACS buffer). The cells were then washed with FACS buffer, blocked with anti-CD16/32 antibody (ThermoFisher Scientific, clone 93, 1 : 100) at 4 °C for 15 minutes, and stained with fluorochrome-conjugated rat anti-mouse antibodies 1 : 150 at 4 °C for 45 minutes. The antibodies, conjugated dyes, and clone numbers were listed as follows: CD45-BV421 (30-F11; 1 : 200), CD11b-FITC (M1/70; 1 : 100), F4/80-PerCP/Cy5.5 (BM8; 1 : 100), CD11c-PE-eFlour610 (N418; 1 : 100), CD3e-PE-eFlour610 (145-2C11; 1 : 100), CD4-APC-H7 (GK1.5; 1 : 100), CD8α-Percp-eFlour710 (53-6.7; 1 : 100), CD86-PECy7 (1 : 100). The cells were finally washed and resuspended in FACS buffer and analyzed on an LSR Fortessa 4-15 flow cytometer.

Immunohistochemistry analysis

To evaluate tumor proliferation after different treatments, 4T1 tumor-bearing mice were established and treated in the same way as described above, but euthanized on the day at the endpoint. The tumors were excised and fixed in 4 % PFA for 48 h and 70 % ethanol for 1 day. The tissues were embedded in paraffin, sectioned and stained for Ki67, γ-H2AX, and TUNEL by Human Tissue Resource Center at the University of Chicago. The slides were scanned on a CRi Pannoramic SCAN 40× whole slide scanner by Integrated Light.

Immunofluorescence microscopy

For immunofluorescence staining of tumor tissues, the tumors were harvested from the treated mice and embedded into OCT blocks (Fisher Healthcare, USA) and frozen at -80°C for cryostat section. The cryo-sections were fixed with 75% acetone + 25% ethanol at -20°C for 10 min, washed with TBST to remove OCT, blocked by 5% FBS in PBS, incubated with PEeFluro610 anti-CD3 (eBioscience, 1 : 100) or FITC anti-Gran B (1 : 500) at 4°C overnight, cell nuclei were stained with DAPI and then observed using CLSM. For detection of GLUT1, the cryo-sections were incubated with GLUT1 antibody (1 : 100) at 4°C overnight and then stained with the Alexa Fluor 488 conjugated secondary antibody (1 : 3000) in 1% BSA containing PBS at RT for 1 hour. The cell nuclei were stained by DAPI and then observed using CLSM.

Hemolysis assay

The red blood cells (RBCs) were collected from BALB/c mice for hemolysis assay. 0.5 mL of blood was diluted with 15 mL of PBS, and RBCs were separated from the serum via centrifugation at 1,200 rpm for 10 min. After washing six times, the RBC suspension was diluted with 15 mL of PBS. 1 mL of RBCs suspension was centrifuged and added with 1 mL of PBS (negative control), deionized water (positive control), or PBS containing Th-DBB and Th-DBB/Dig. After 12 hours of incubation at 37 ºC, the mixture was centrifuged at 14,500 rpm for 10 min, and 100 μL of supernatant was transferred to a 96-well plate. The absorbance at 540 nm of supernatant was detected by the HTX microplate reader. The hemolysis ratios were calculated based on the equation: Hemolysis (%) = (sample absorbance − negative control absorbance)/(positive control absorbance − negative control absorbance) × 100%.

Statistical analysis

All the statistical analysis was performed on Origin Lab software. The experimental results were presented with average values, and expressed as the mean ± SD. P value was calculated with Microsoft Excel software by student's two-tailed t-test.

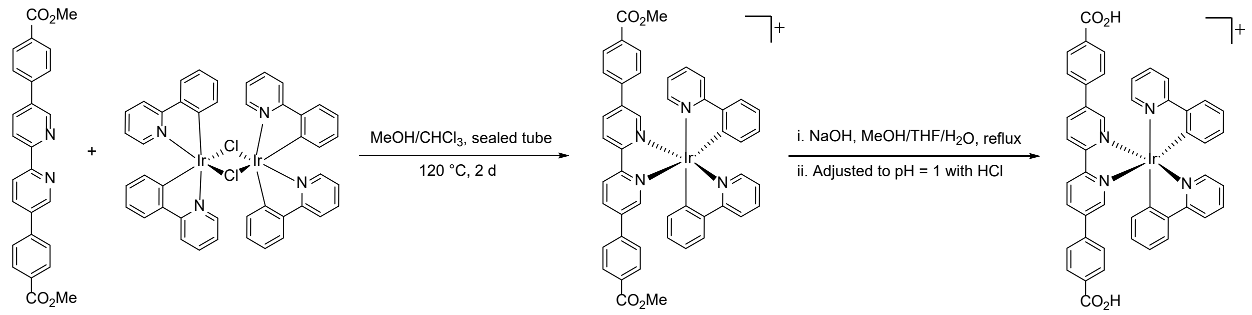

Scheme S1. Synthesis of Ir-DBB.

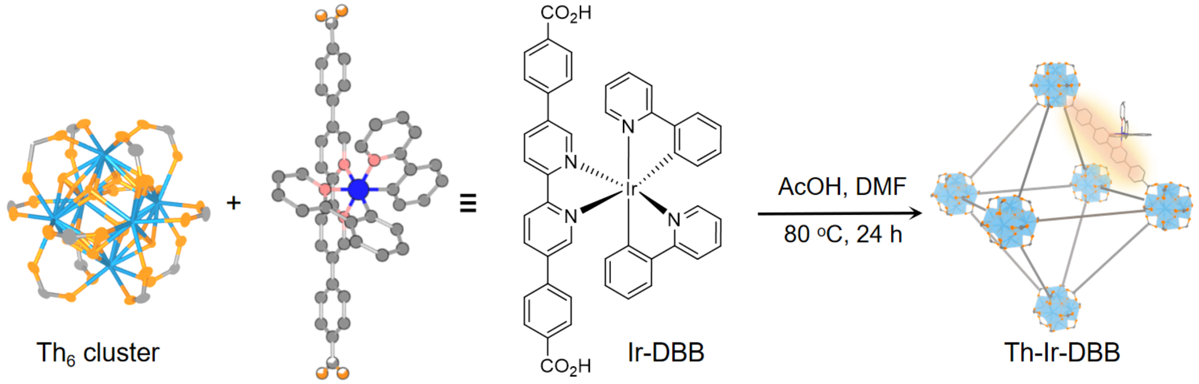

Scheme S2. Synthesis of Th-Ir-DBB.

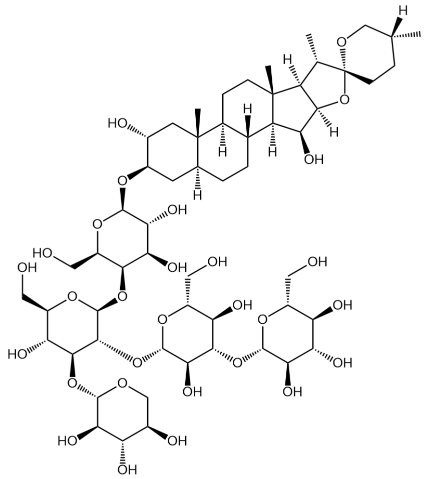

Scheme S3. Chemical Structure of digitonin.

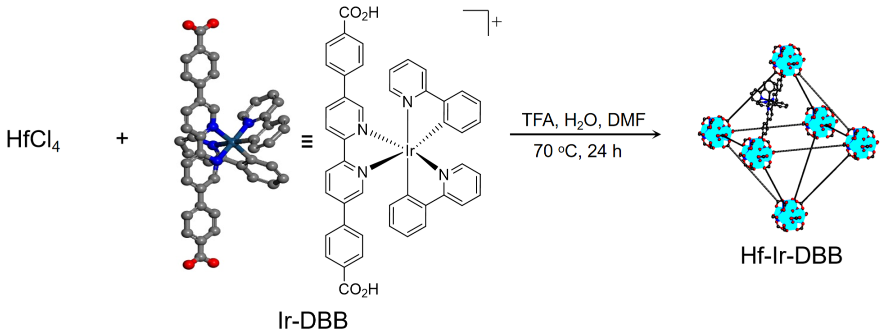

Scheme S4. Synthesis of Hf-Ir-DBB.

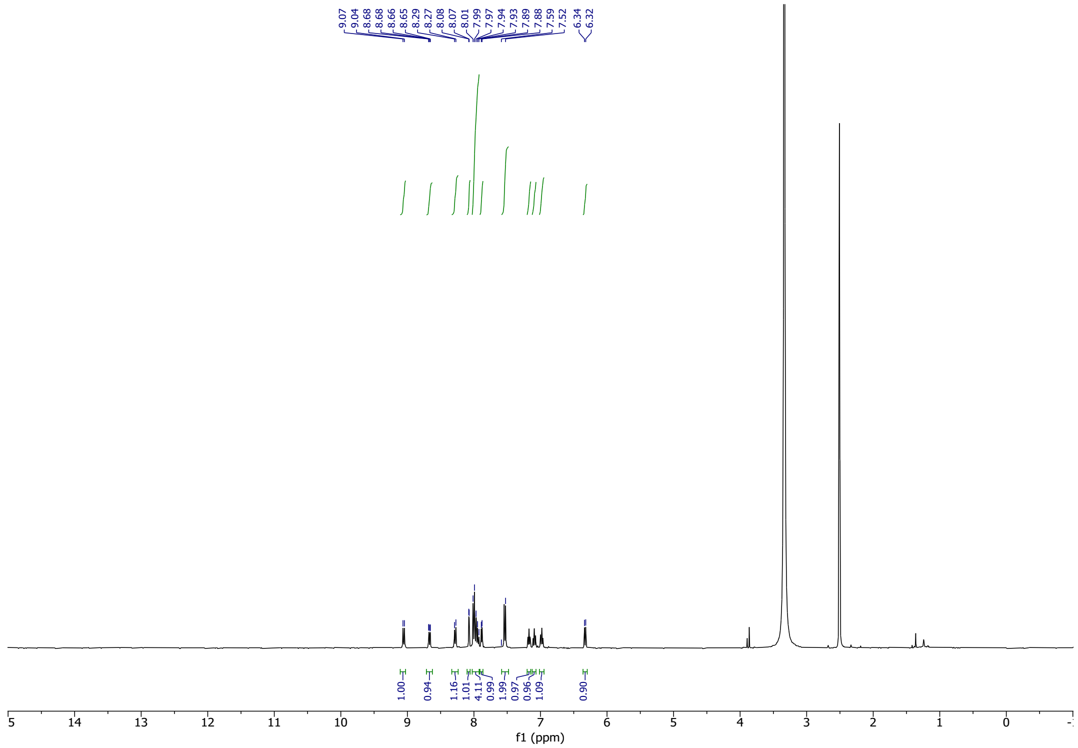

Figure S1. ^{1}H NMR spectrum of Ir-DBB.

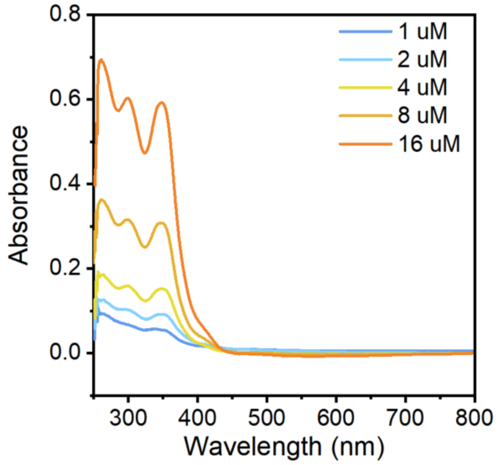

Figure S2. UV-Vis spectra of Ir-DBB at different concentrations in DMSO.

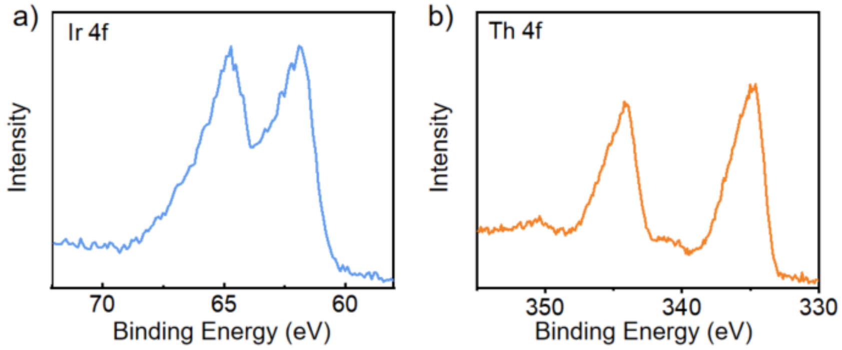

Figure S3. X-ray photoelectron spectroscopy (XPS) spectra in the (a) Ir 4f and (b) Th 4f regions.

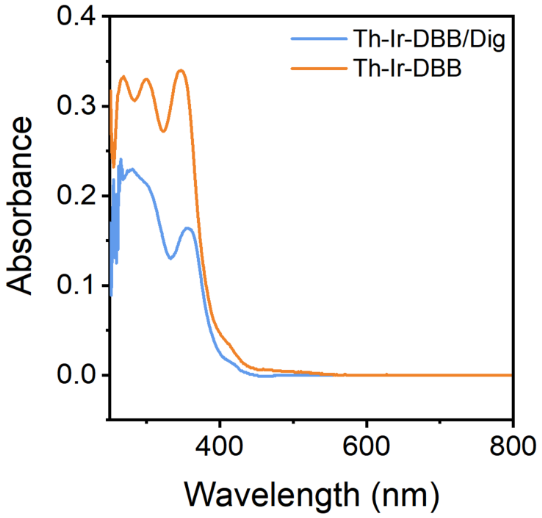

Figure S4. UV-Vis spectra of digested Th-Ir-DBB and Th-Ir-DBB/Dig in DMSO.

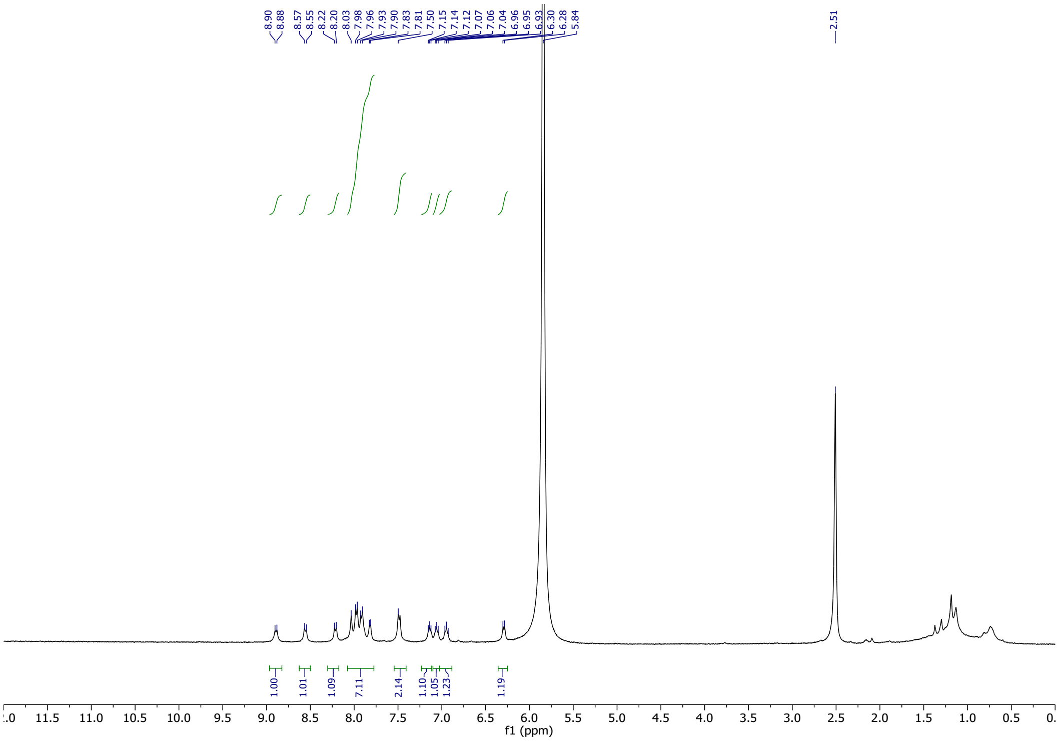

Figure S5. ^{1}H NMR spectrum of digested Th-Ir-DBB.

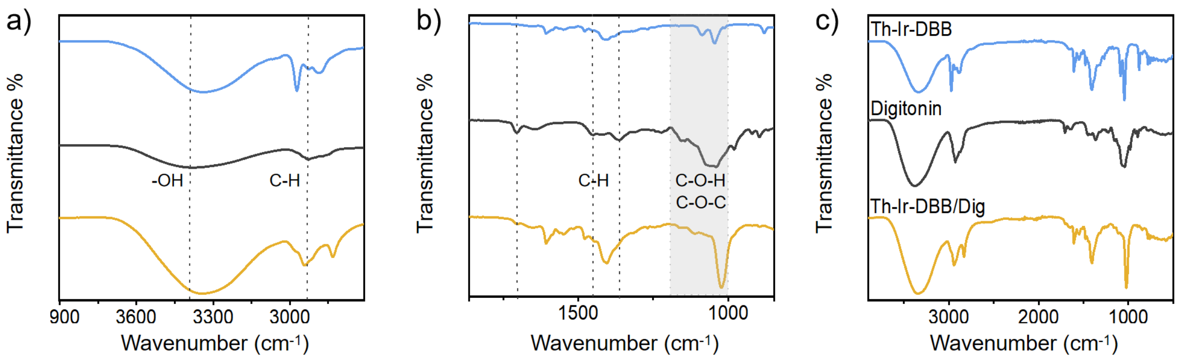

Figure S6. FT-IR spectra of Th-Ir-DBB, digitonin, and Th-Ir-DBB/Dig.

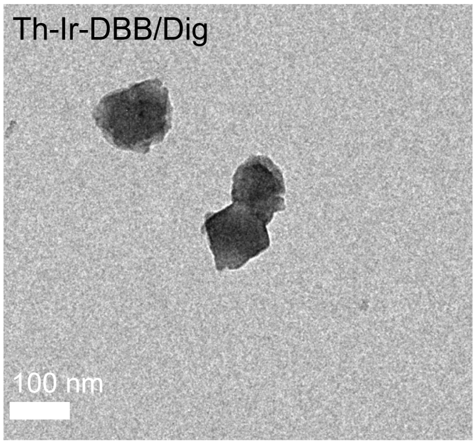

Figure S7. TEM image of Th-Ir-DBB/Dig (zoomed-in TEM image in Figure 1f).

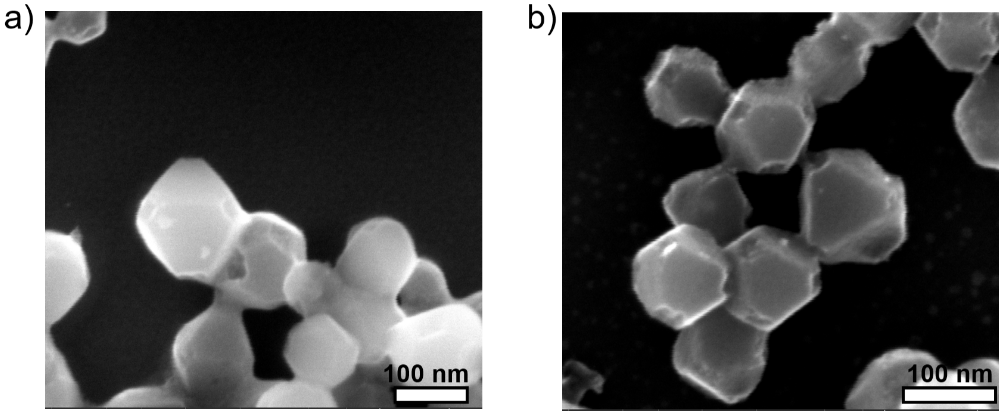

Figure S8. Scanning electron microscope (SEM) images of Th-Ir-DBB (a) and Th-Ir-DBB/Dig (b).

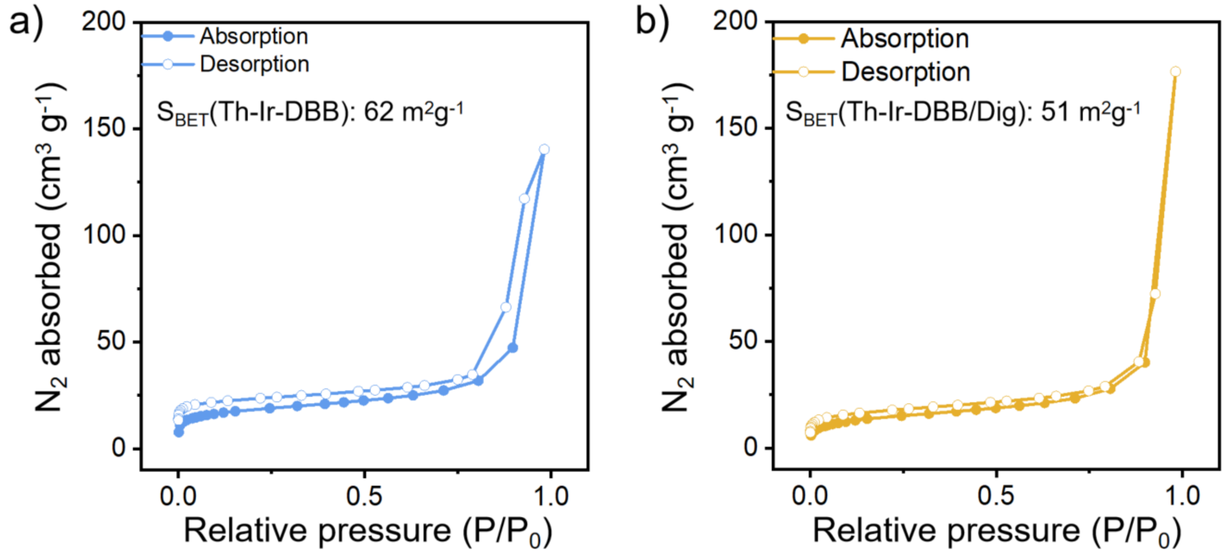

Figure S9. N_2 adsorption isotherms of Th-Ir-DBB (a) and Th-Ir-DBB/Dig (b) at 77 K.

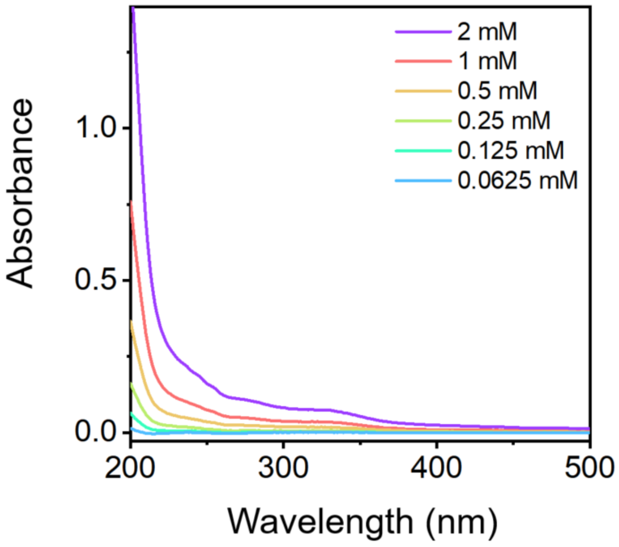

Figure S10. Standard curve of digitonin determined by UV-Vis.

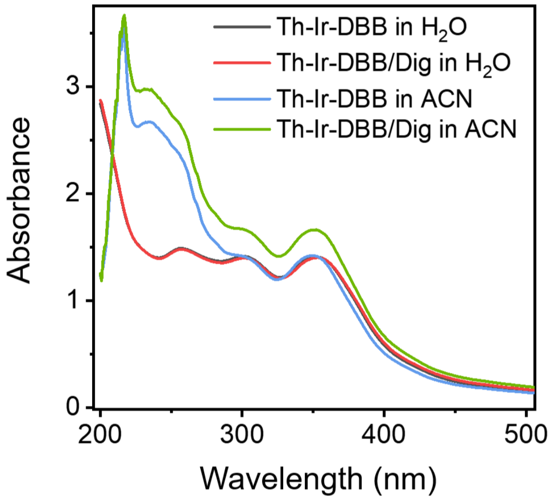

Figure S11. UV-Vis spectra of Th-Ir-DBB and Th-Ir-DBB/Dig in water or ACN.

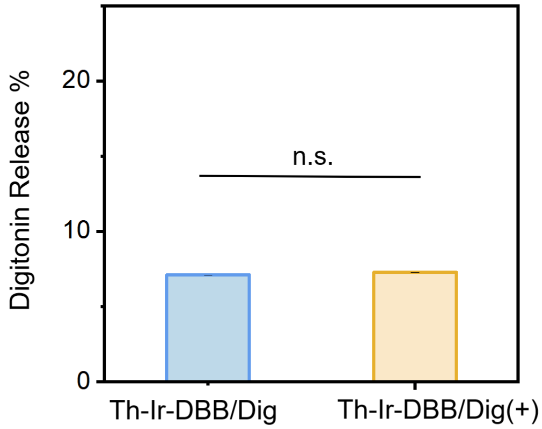

Figure S12. Release ratios of digitonin from aqueous dispersions of Th-Ir-DBB/Dig with or without X-ray irradiation (6 Gy, n = 3).

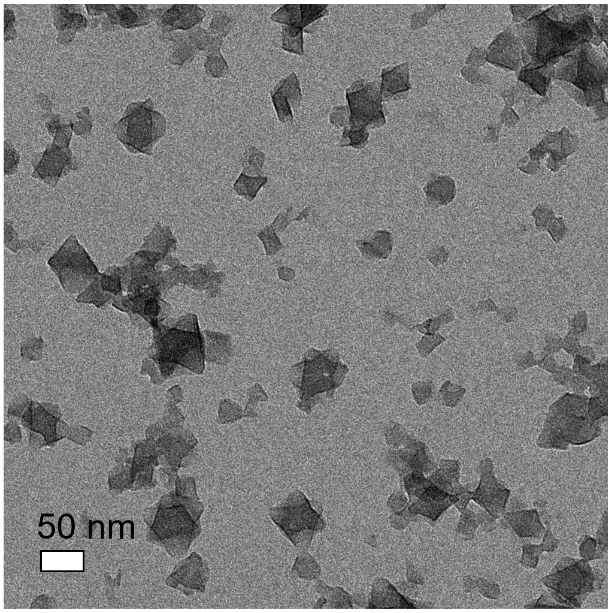

Figure S13. TEM image of Th-Ir-DBB/Dig after incubation in PBS for 6 h (pH 7.4, 1 mM).

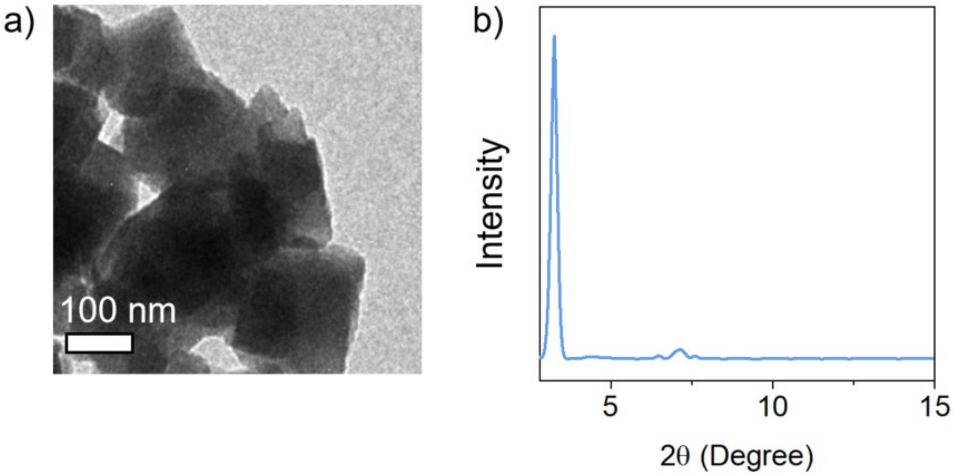

Figure S14. (a) TEM image of Hf-Ir-DBB. (b) XRD pattern of Hf-Ir-DBB.

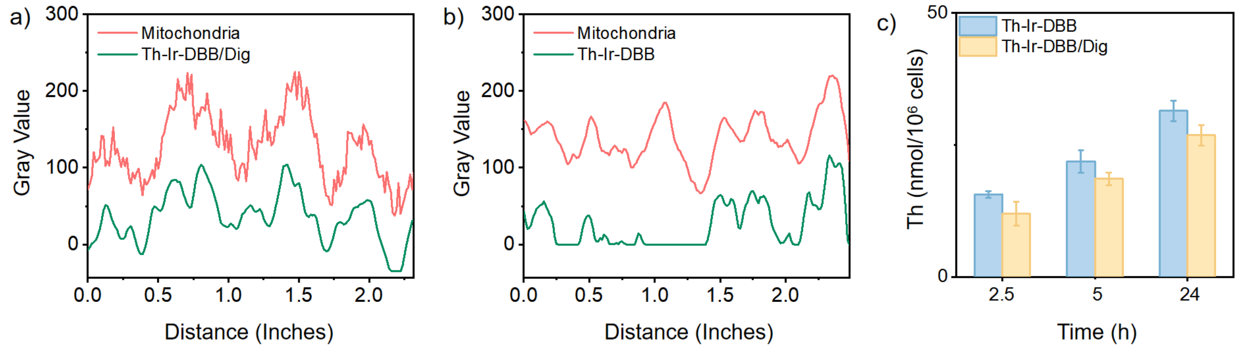

Figure S15. Line scan analysis of relative fluorescence intensity of mitochondria and Th-Ir-DBB/Dig (a) or Th-Ir-DBB (b) studied by Image J software. (c) Time-dependent enrichment of Th-Ir-DBB and Th-Ir-DBB/Dig in mitochondria as measured by ICP-MS (n = 3).

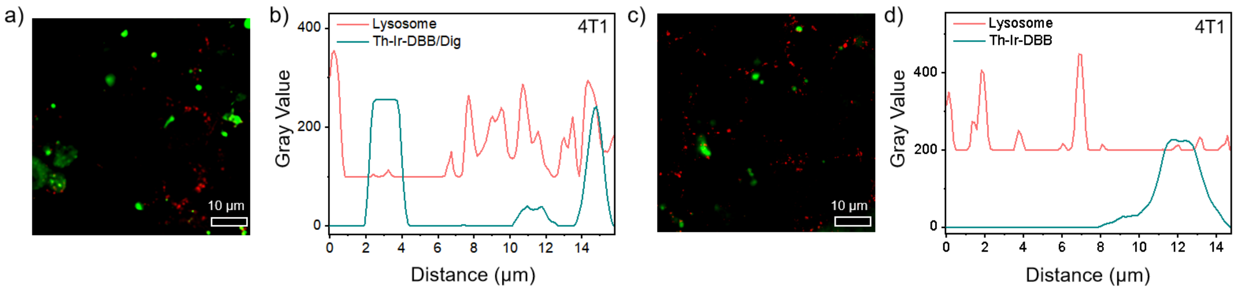

Figure S16. CLSM images of lysosomes in 4T1 cells treated with Th-Ir-DBB/Dig (a) or Th-Ir-DBB (c). Line scan analysis of relative fluorescence intensity of lysosome (red) and Th-Ir-DBB/Dig (c) or Th-Ir-DBB (d) studied with Image J software.

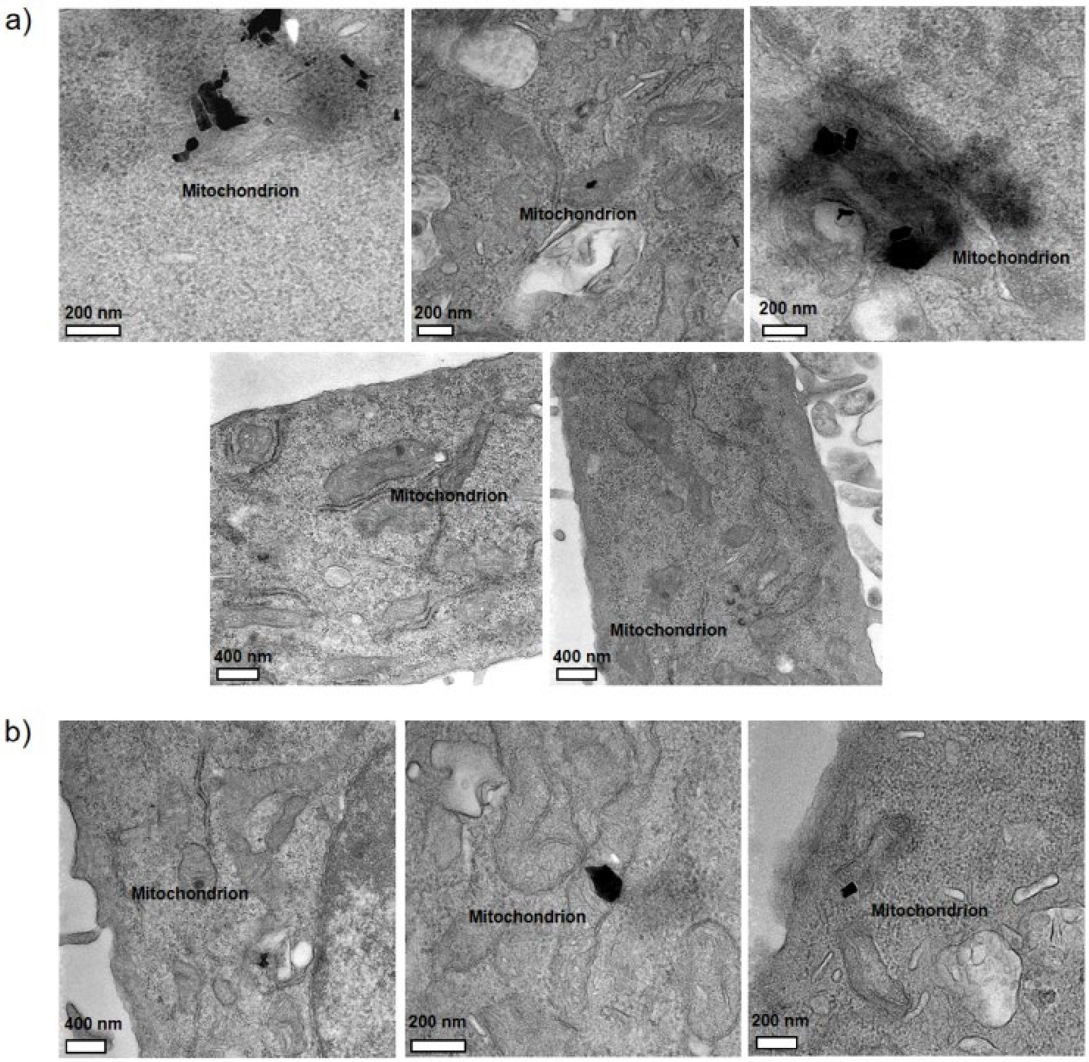

Figure S17. Accumulation of Th-Ir-DBB (Th: 20 μM, a) Th-Ir-DBB/Dig (b) in 4T1 cells after 4-hour incubation as observed by TEM.

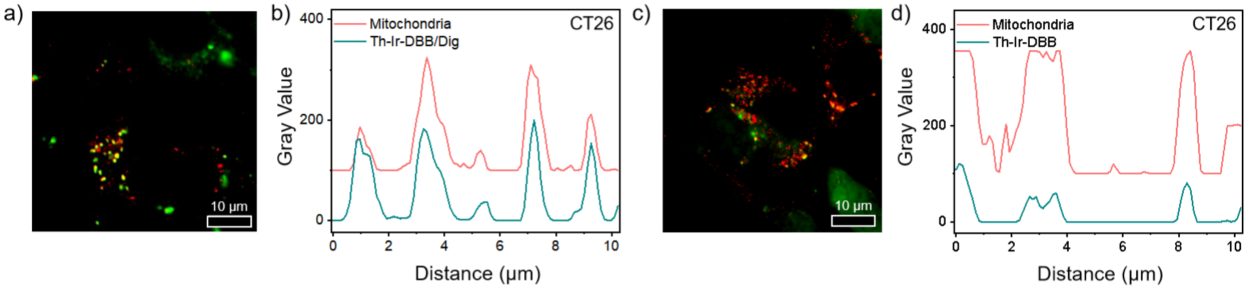

Figure S18. CLSM images of mitochondria in CT26 cells treated with Th-Ir-DBB/Dig (a) or Th-Ir-DBB (c). Line scan analysis of relative fluorescence intensity of mitochondria (red) and Th-Ir-DBB/Dig (c) or Th-Ir-DBB (d) studied with Image J software.

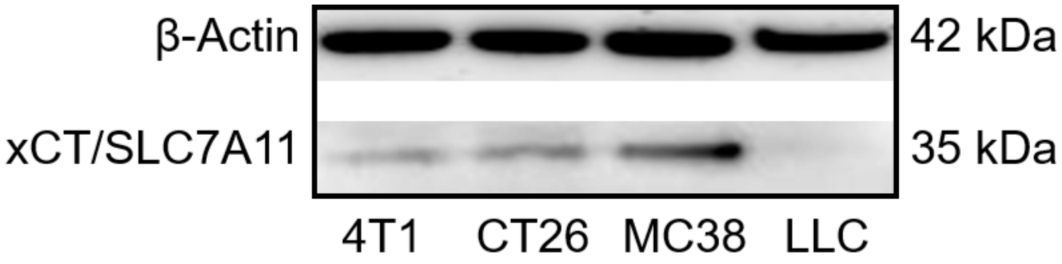

Figure S19. Western blot results of xCT/SLC7A11 levels in 4T1, CT26, MC38 and LLC cells.

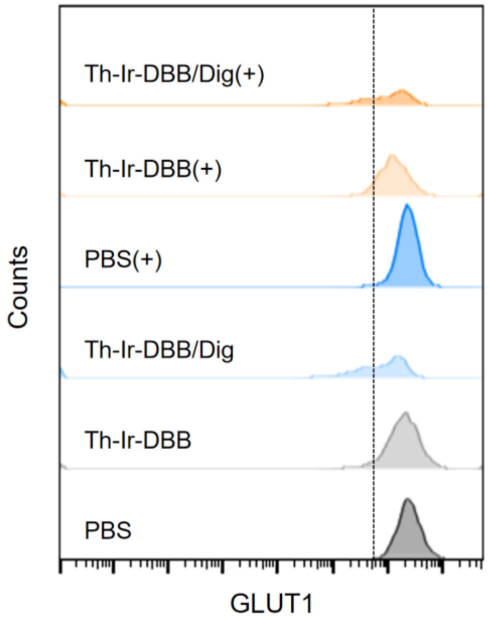

Figure S20. GLUT1 expression levels of in treated 4T1 cells detected by flow cytometry.

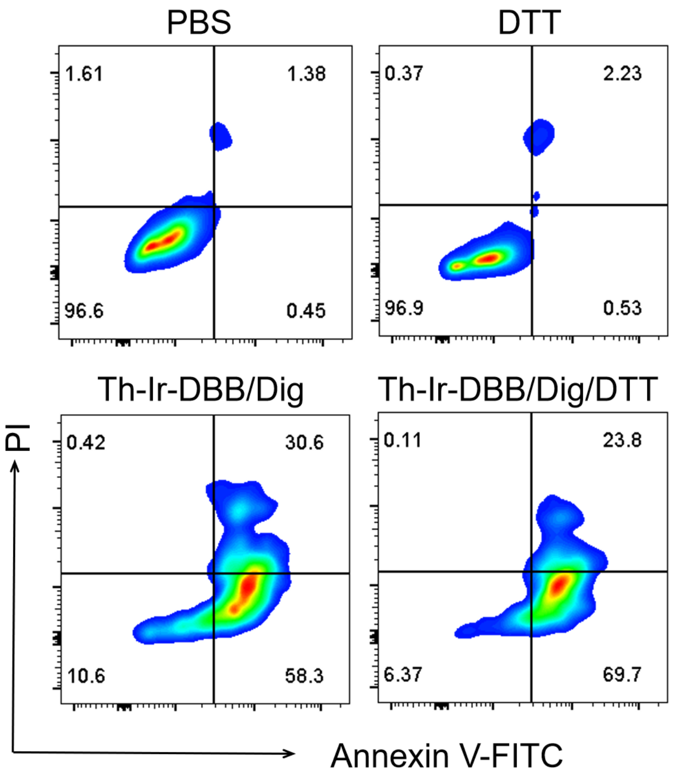

Figure S21. Apoptosis assay using flow cytometry after staining with annexin V-FITC/propidium iodide (PI).

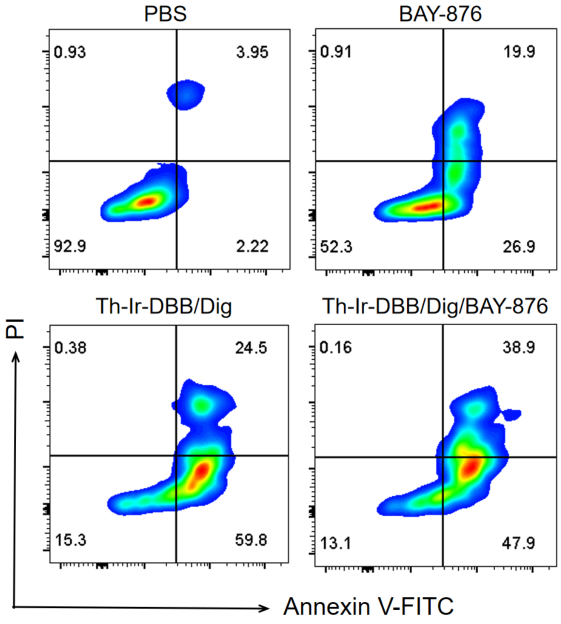

Figure S22. Apoptosis assay using flow cytometry after staining with annexin V-FITC/propidium iodide (PI).

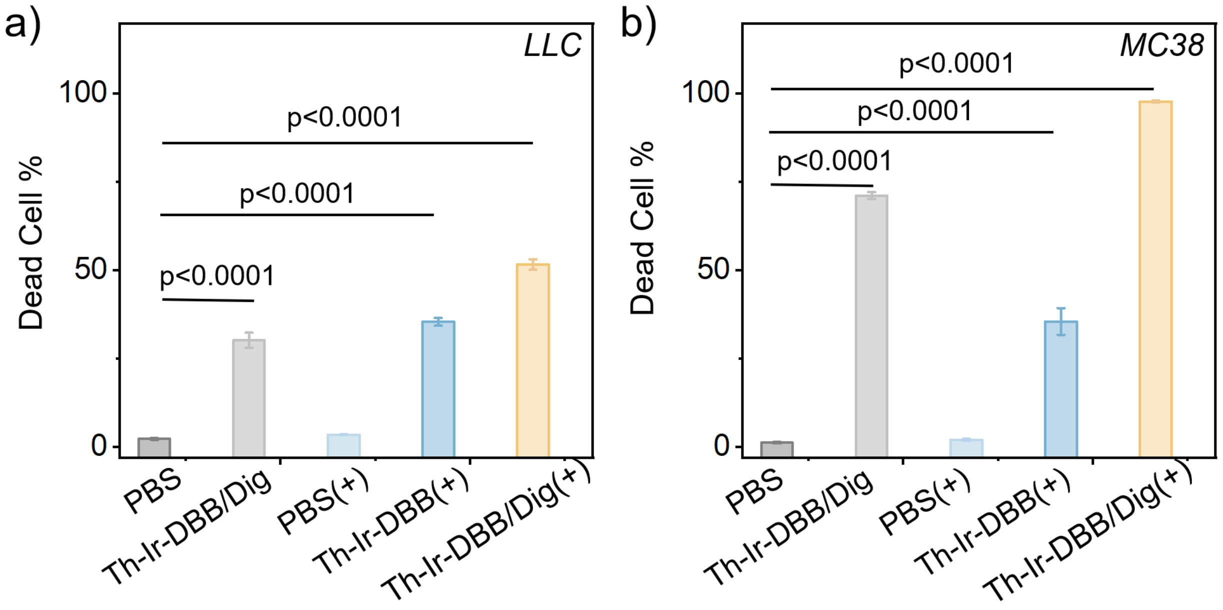

Figure S23. Percentages of dead (late apoptotic and necrotic) LLC cells (a) or MC38 cells (b) after different treatments (n = 3).

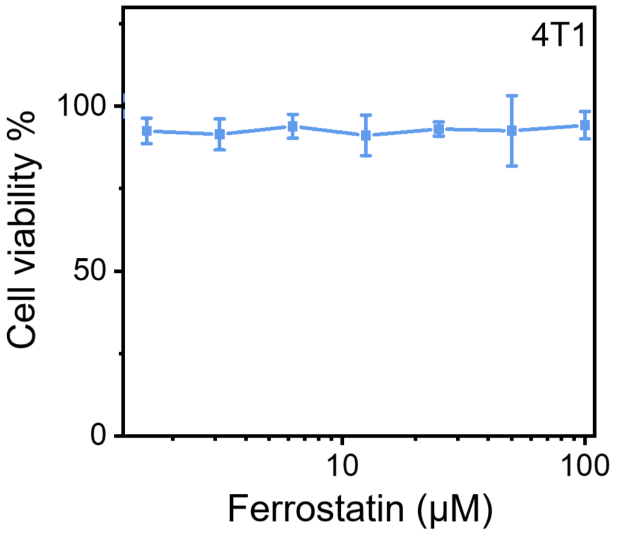

Figure S24. Viability of 4T1 cells after incubation with different concentrations of the ferroptosis inhibitor ferrostatin for 48 hours.

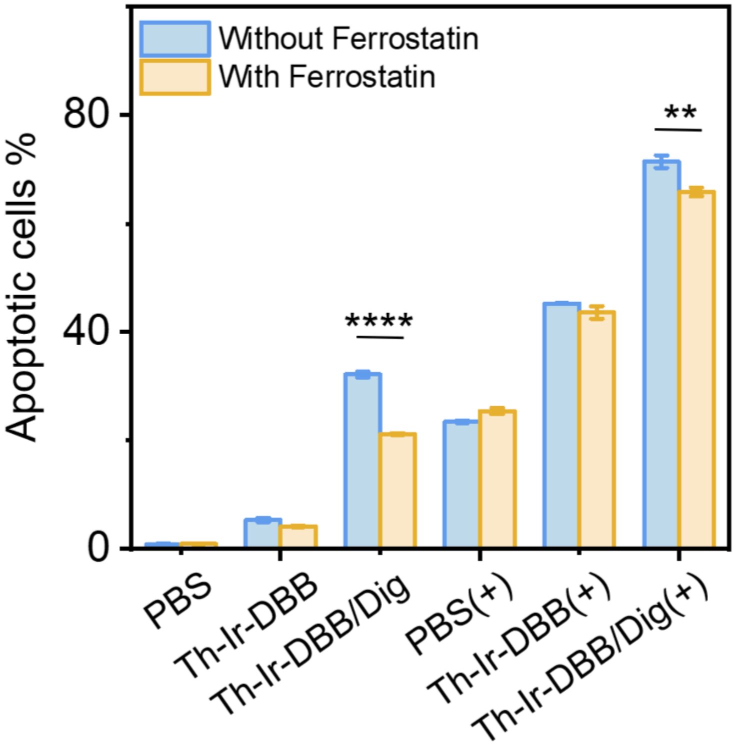

Figure S25. Percentages of apoptotic 4T1 cells after different treatments (n = 3).

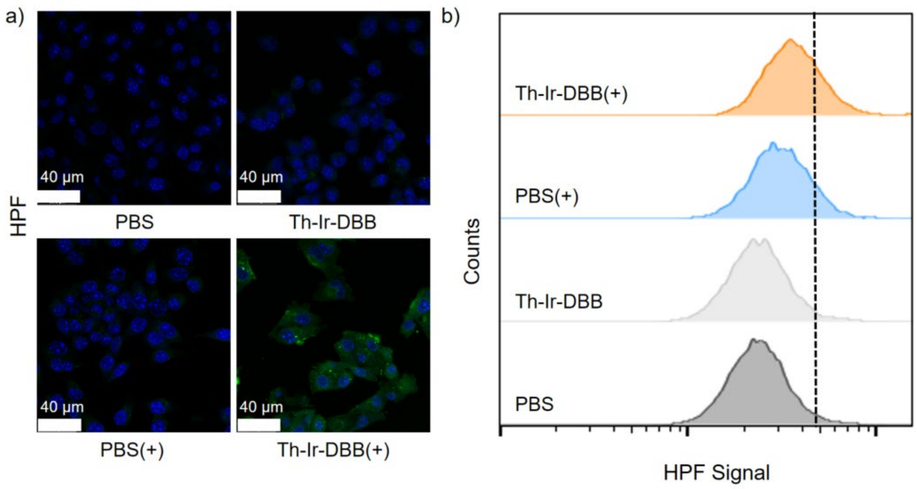

Figure S26. (a) · OH levels of in treated 4T1 cells probed by HPF as observed by CLSM. (b) ·OH levels of in treated 4T1 cells probed by HPF as detected by flow cytometry.

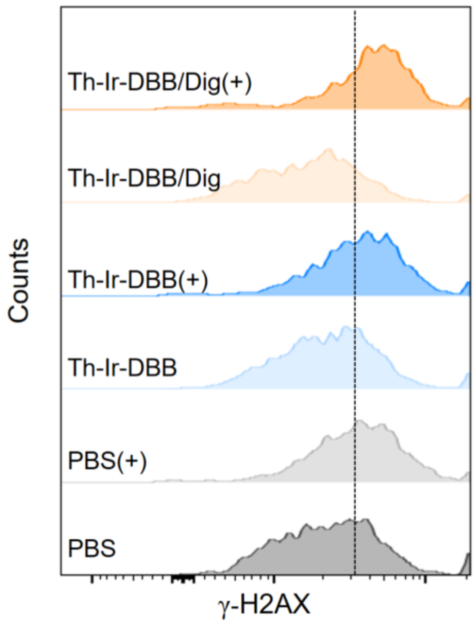

Figure S27. γ-H2AX expression levels of in treated 4T1 cells detected by flow cytometry.

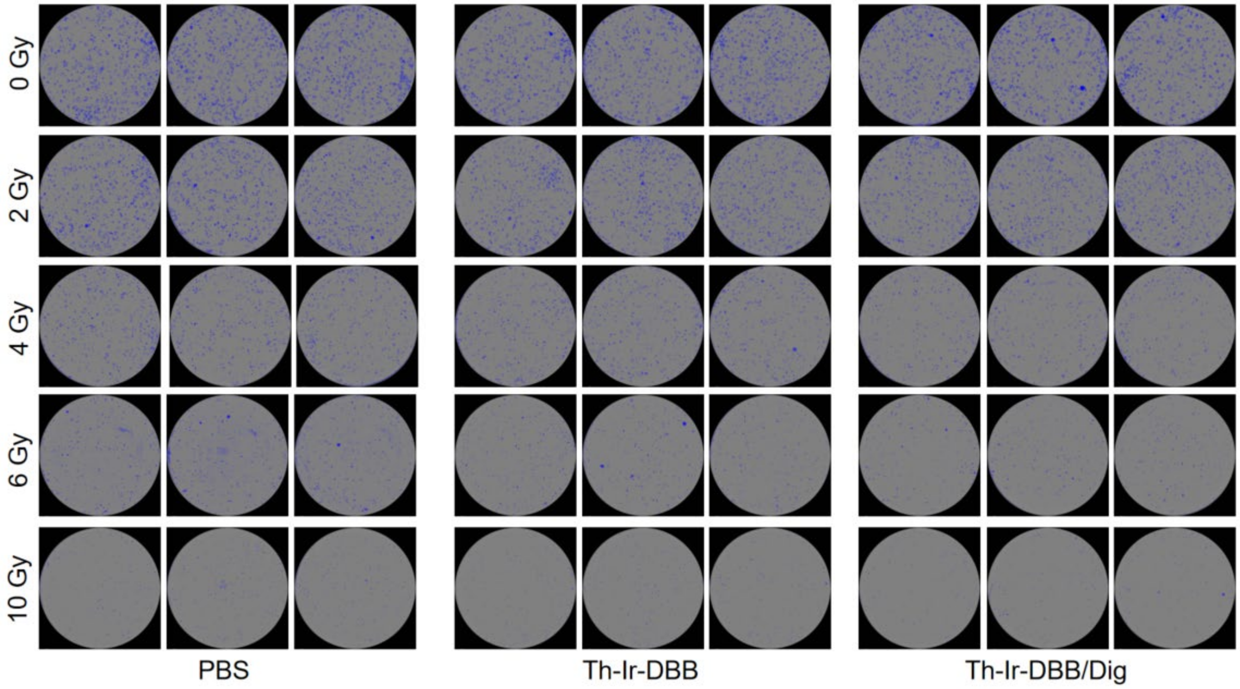

Figure S28. Representative images of colonies formed by treated CT26 cells (well diameter = 35 mm).

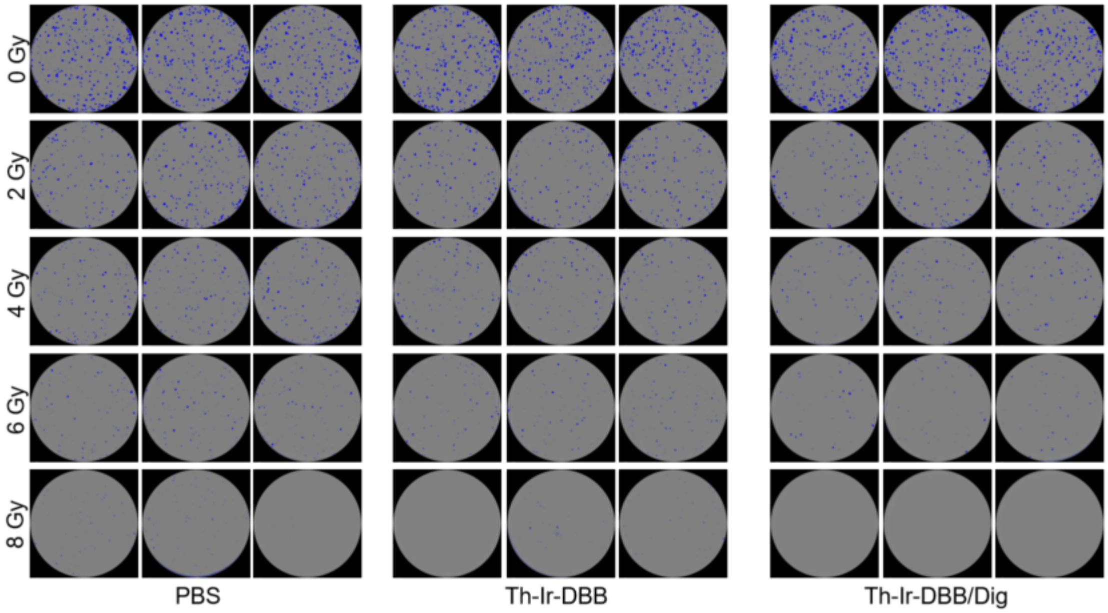

Figure S29. Representative images of colonies formed by treated 4T1 cells (well diameter = 35 mm).

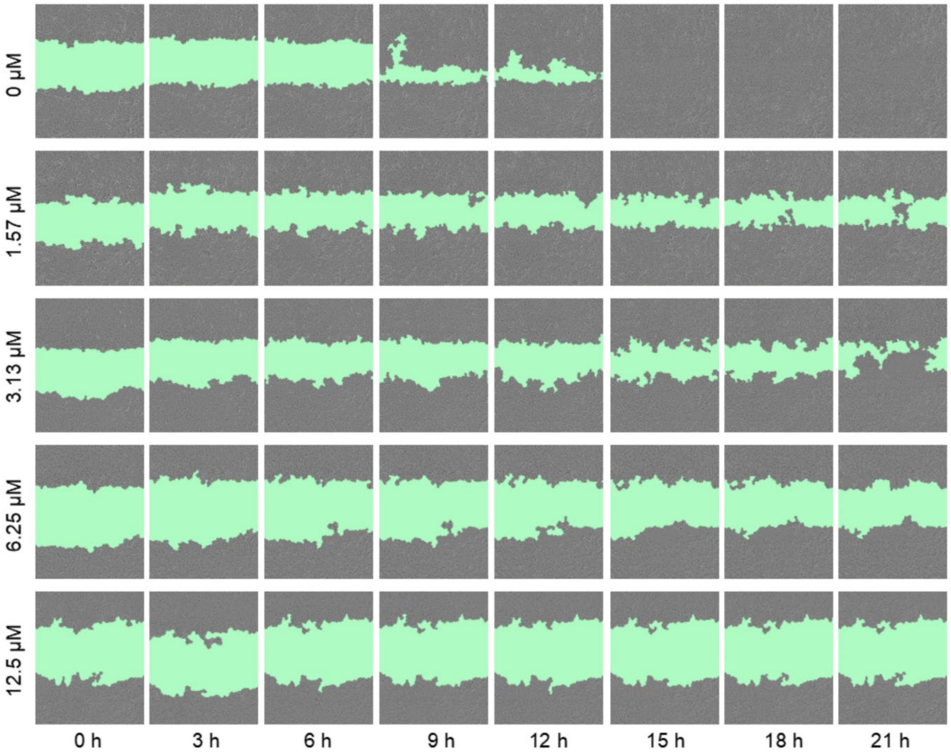

Figure S30. Time-dependent scratch wound assays of 4T1 cells after being incubated with digitonin at different concentrations.

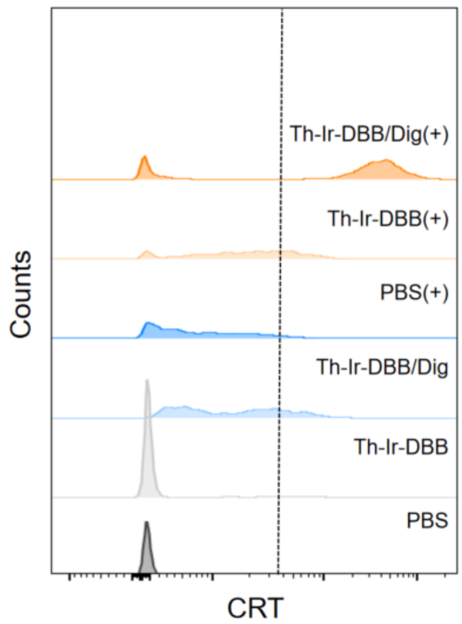

Figure S31. CRT expression levels in treated 4T1 cells detected by flow cytometry.

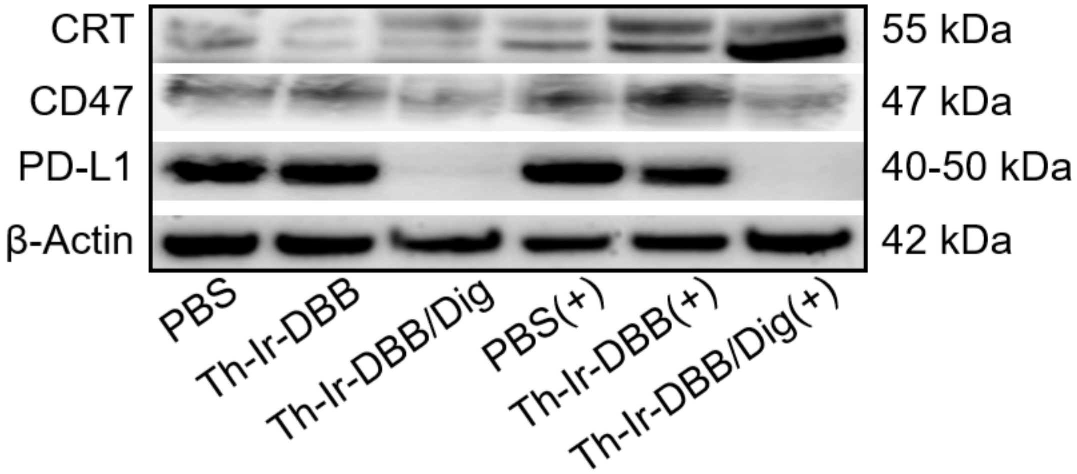

Figure S32. Western blot results of CRT, CD47, PD-L1 and β-actin levels in 4T1 cells after different treatments.

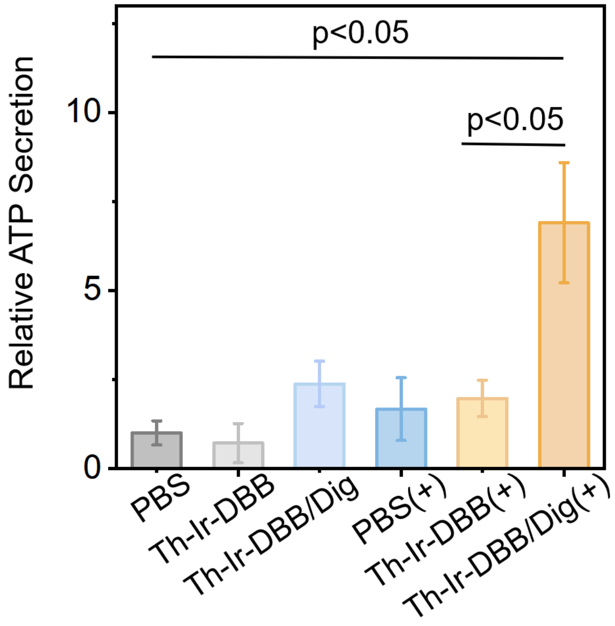

Figure S33. Relative secreted ATP contents in the supernatants of 4T1 cells after different treatments.

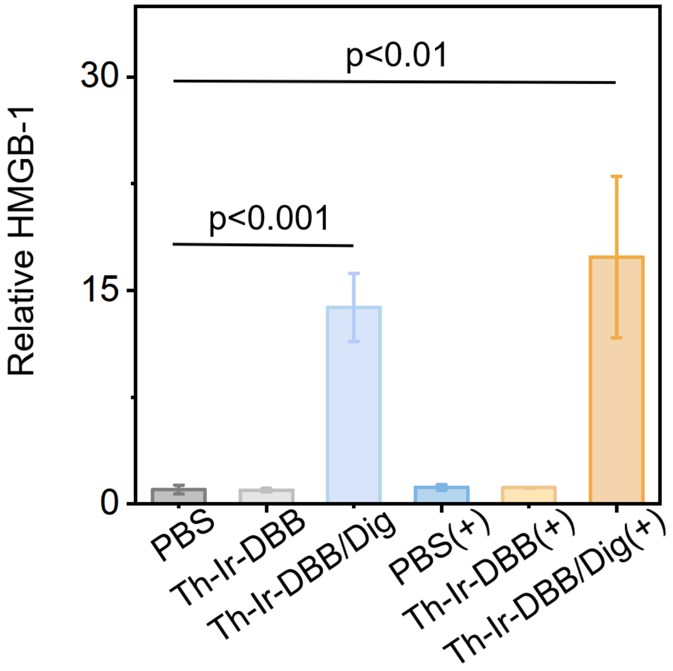

Figure S34. Relative secreted HMGB-1 contents in the supernatants of 4T1 cells after different treatments.

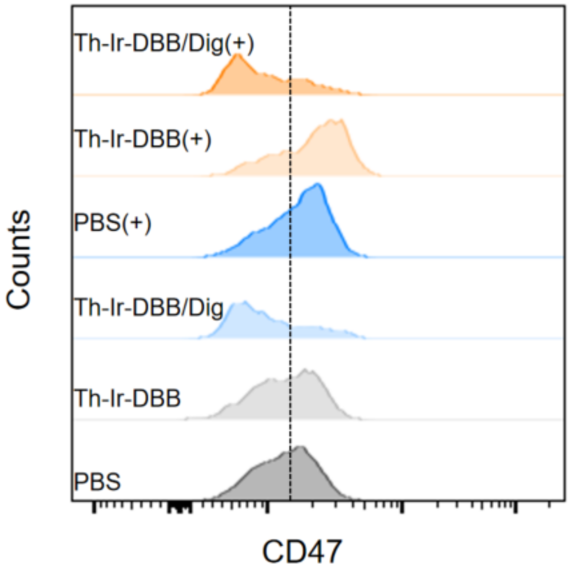

Figure S35. CD47 levels in treated 4T1 cells detected by flow cytometry.

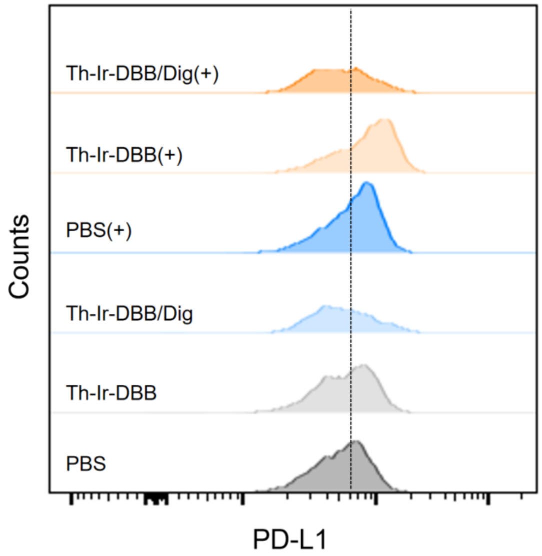

Figure S36. PD-L1 levels in treated 4T1 cells detected by flow cytometry.

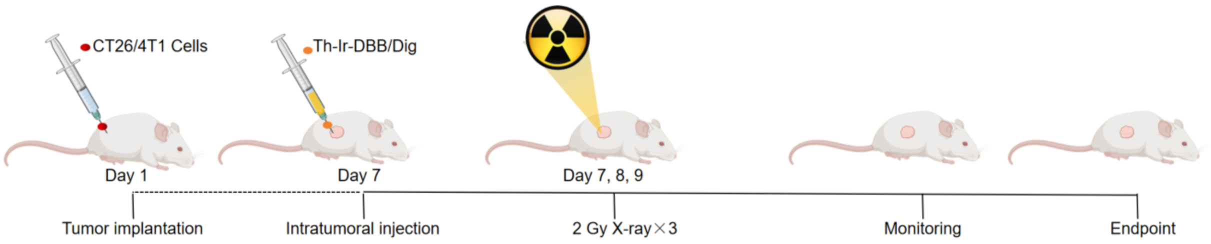

Figure S37. Schematic illustration of the dosing schedule on subcutaneous 4T1 or CT26 tumor models in BALB/c mice.

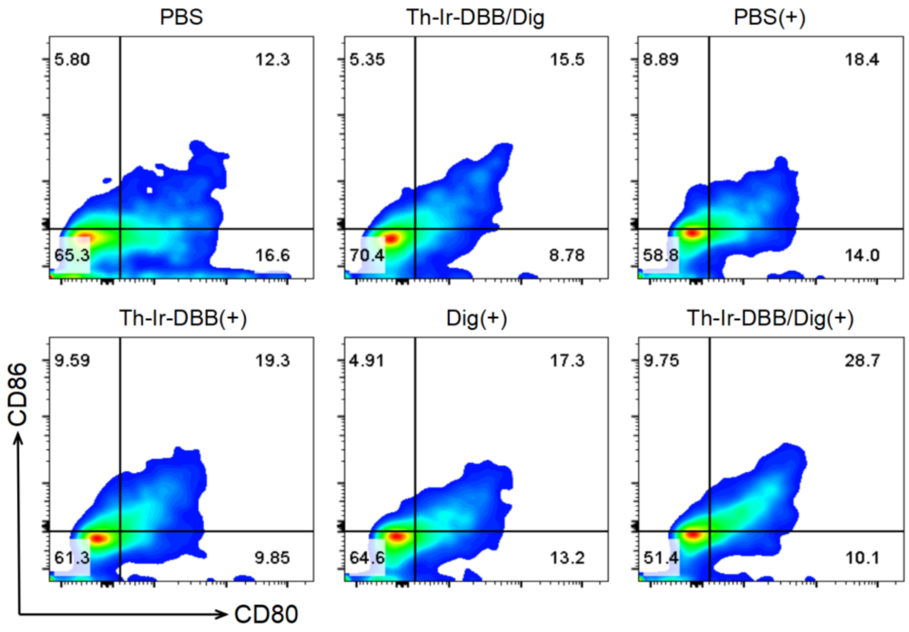

Figure S38. Representative mature DCs ($CD80^+$ $CD86^+$) in $CD11c^+$ cells after different treatments.

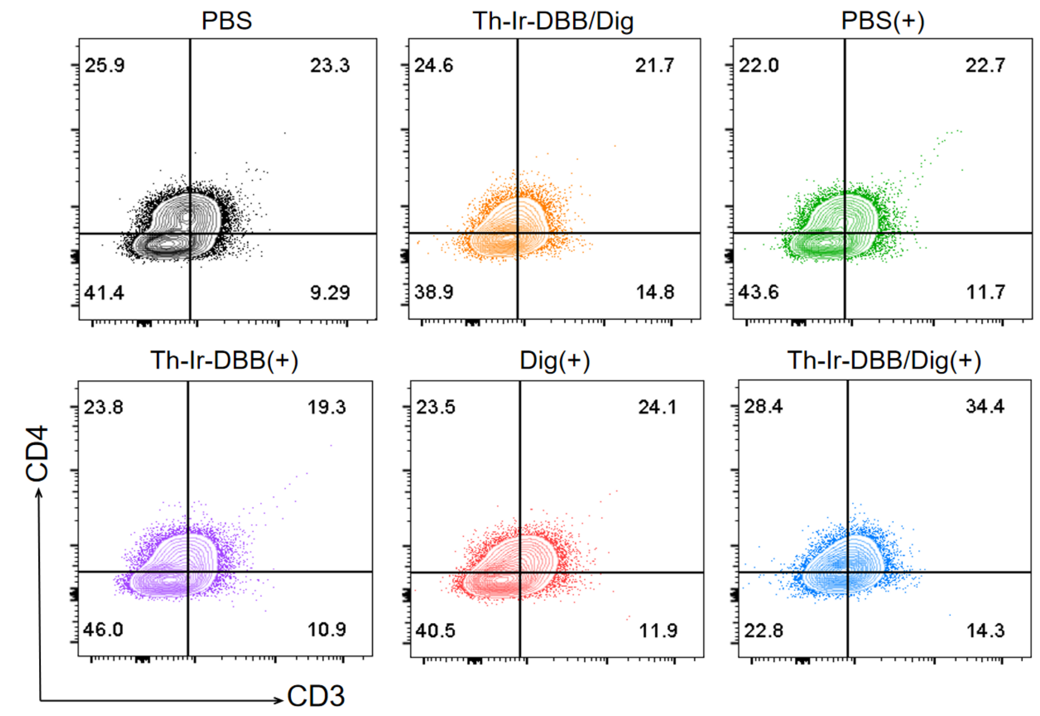

Figure S39. Representative helper T cells ($CD3^{+}CD4^{+}$) in $CD45^{+}$ cells after different treatments.

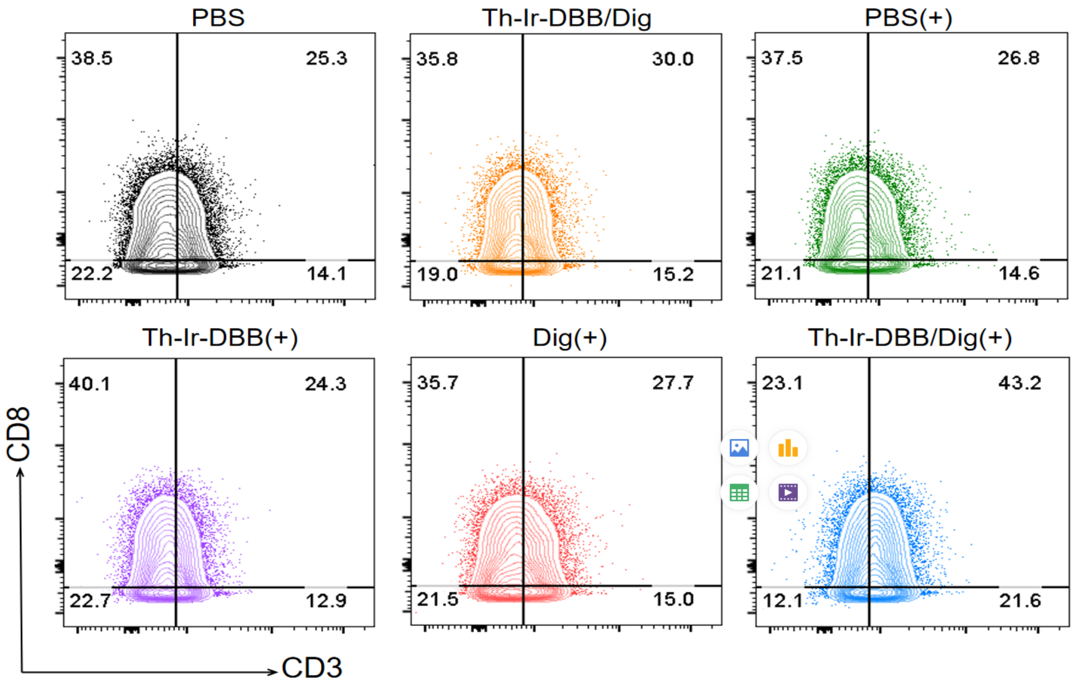

Figure S40. Representative cytotoxic T cells ($CD3^{+}CD8a^{+}$) in $CD45^{+}$ cells after different treatments.

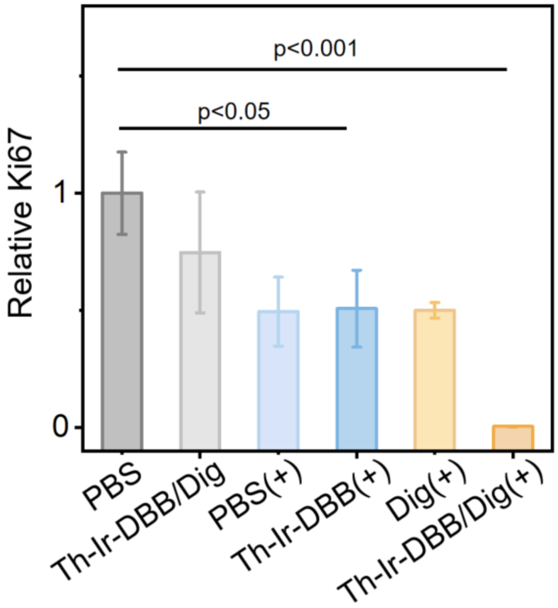

Figure S41. Relative $Ki67^+$ cells in the 4T1 tumor tissues after different treatments.

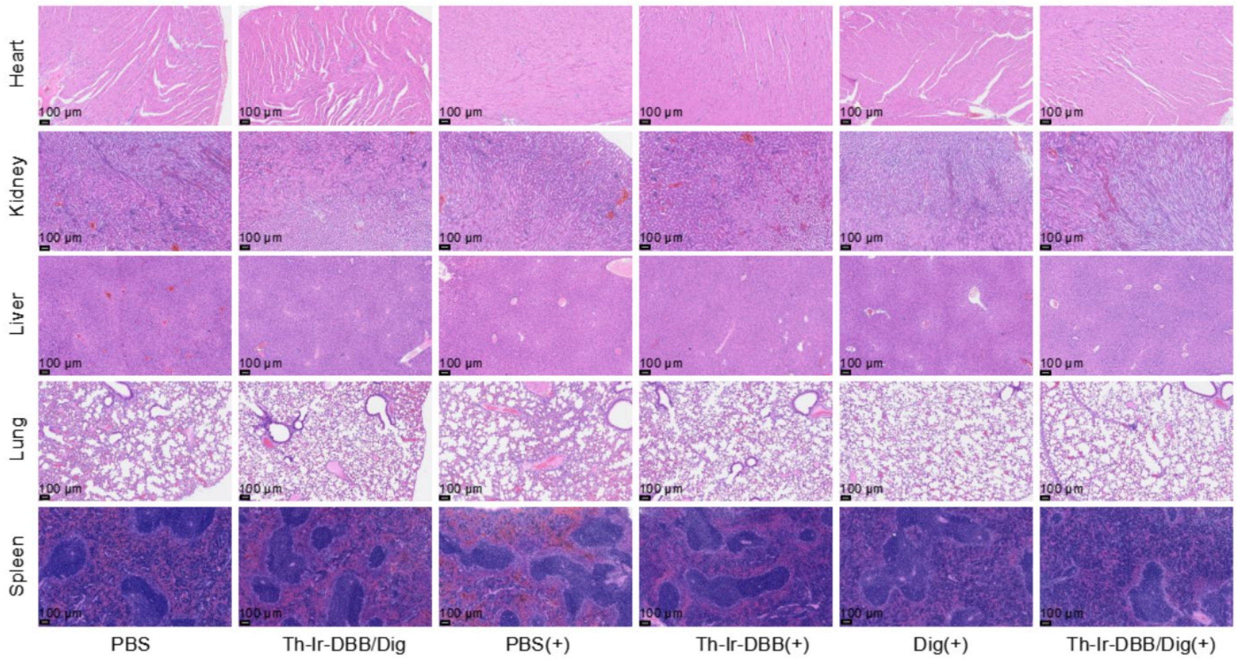

Figure S42. H&E staining of normal organs (heart, kidney, liver, lung and spleen) of 4T1 tumor-bearing BALB/c mice after different treatments.

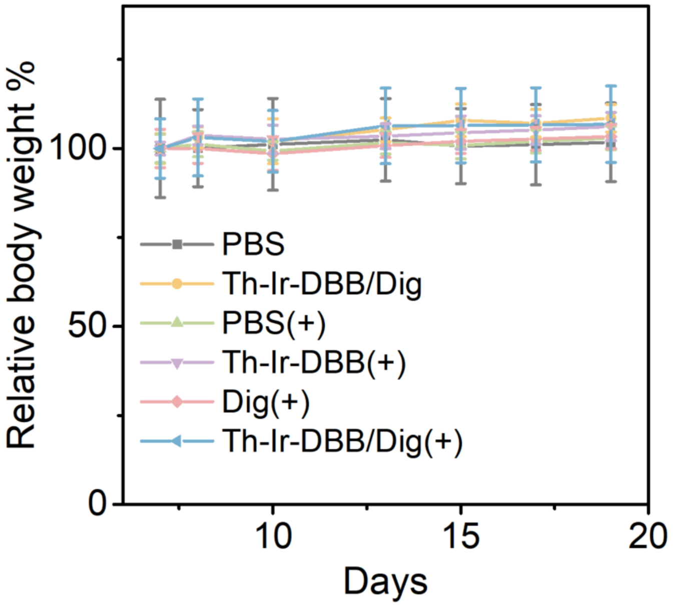

Figure S43. Relative body weight of CT26 tumor-bearing BALB/c mice (n = 5).

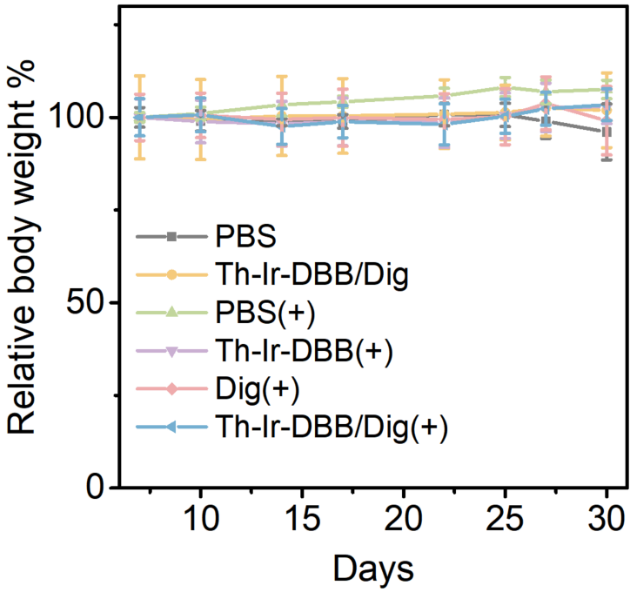

Figure S44. Relative body weight of 4T1 tumor-bearing BALB/c mice (n = 5).

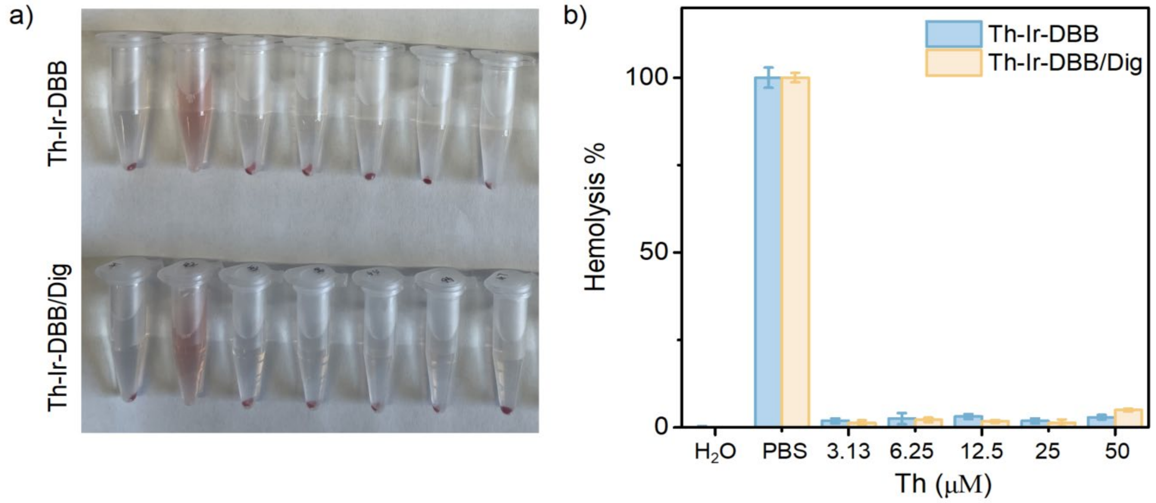

Figure S45. (a) Images of centrifuged blood cells after being incubated with Th-Ir-DBB or Th-Ir-DBB/Dig with different concentrations for 12 hours at 37 ºC. (b) Hemolysis assay of Th-Ir-DBB or Th-Ir-DBB/Dig after being incubated with red cells for 12 h at 37ºC.

Table S1. TGI values of subcutaneous CT26 tumors and 4T1 tumors at the endpoints.

Treatment	TGI (CT26)	TGI (4T1)
Th-Ir-DBB/Dig	14.12%	23.14%
PBS(+)	68.45%	33.3%
Th-Ir-DBB(+)	85.45%	56.84%
Dig(+)	73.45%	38.13%
Th-Ir-DBB/Dig(+)	97.68%	86.12%

References

[1] K. E. Knope, R. E. Wilson, M. Vasiliu, D. A. Dixon, L. Soderholm, *Inorganic Chemistry* **2011**, 50, 9696.
[2] G. Lan, K. Ni, S. S. Veroneau, T. Luo, E. You, W. Lin, *Journal of the American Chemical Society* **2019**, 141, 6859.
